# Lead-Free Perovskite Materials for Solar Cells

**DOI:** 10.1007/s40820-020-00578-z

**Published:** 2021-01-25

**Authors:** Minghao Wang, Wei Wang, Ben Ma, Wei Shen, Lihui Liu, Kun Cao, Shufen Chen, Wei Huang

**Affiliations:** 1 Key Laboratory for Organic Electronics and Information Displays & Jiangsu Key Laboratory for Biosensors, Institute of Advanced Materials (IAM), Jiangsu National Synergetic Innovation Center for Advanced Materials (SICAM), Nanjing University of Posts & Telecommunications (NUPT), 9 Wenyuan Road, Nanjing, 210023 People’s Republic of China; 2 Frontiers Science Center for Flexible Electronics, Xi’an Institute of Flexible Electronics (IFE) and Xi’an Institute of Biomedical Materials & Engineering, Northwestern Polytechnical University, 127 West Youyi Road, Xi’an, 710072 People’s Republic of China

**Keywords:** Solar cells, Perovskite, Lead-free, First-principles calculation, Photovoltaic

## Abstract

The toxicity issue of lead hinders large-scale commercial production and photovoltaic field application of lead halide perovskites. Some novel non- or low-toxic perovskite materials have been explored for development of environmentally friendly lead-free perovskite solar cells (PSCs). This review studies the substitution of equivalent/heterovalent metals for Pb based on first-principles calculation, summarizes the theoretical basis of lead-free perovskites, and screens out some promising lead-free candidates with suitable bandgap, optical, and electrical properties. Then, it reports notable achievements for the experimental studies of lead-free perovskites to date, including the crystal structure and material bandgap for all of lead-free materials and photovoltaic performance and stability for corresponding devices. The review finally discusses challenges facing the successful development and commercialization of lead-free PSCs and predicts the prospect of lead-free PSCs in the future.

## Highlights

- The toxicity issue of lead-based halide perovskites hinders theirs large-scale commercial applications in solar cells.
- A variety of non- or low-toxic perovskite materials have been used for development of environmentally friendly lead-free perovskite solar cells, some of which show excellent optoelectronic properties and device performances.
- At present, more new lead-free perovskite materials with tunable optical and electrical properties are urgently required to design highly efficient and stable lead-free perovskite solar cells.

## Graphical abstract

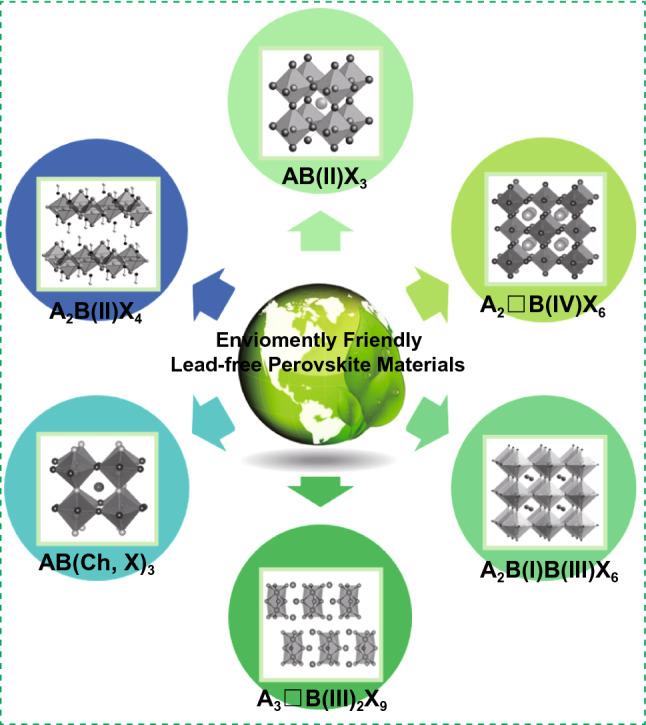

## Introduction

As a major driving force of the world today, traditional fossil fuels have been essential from the first industrial revolution. Although previous industrial revolutions have brought human society into an unprecedented boom era, they have simultaneously caused tremendous energy and resource consumption and aggravated the contradiction between human and nature, making us have to pay a huge environmental price and ecological cost. With the fast development of world’s economy and social productive forces, the requirement of energy has also been growing rapidly and the traditional fossil energy has been incapable of meeting our demands. So entering the twenty-first century, humanity has been facing unprecedented challenges of global crises of energy and resource, environment, ecology, and climate change. All of these issues have initiated the fourth industrial revolution, namely the green industrial revolution. At this time, solar energy as the initial source of all energy inevitably becomes new support for human and social development due to its advantages of pollution-free, renewability, huge energy, and so on. Optoelectronic conversion as one of the major utilizations of solar energy has been widely researched in the past several decades, and solar cells belong to the most important type of optoelectronic converters. The development of low-cost, high-efficiency solar cells has become a central issue in recent years. And perovskite solar cells (PSCs), as a promising class of solar cells family, have attracted intensive attention in the past decade due to high absorption coefficient, excellent bipolar charge mobility, long carrier diffusion length, low exciton binding energy, low trap state density, and tunable bandgap.

The power conversion efficiency (PCE) of PSCs based on lead (Pb) perovskites has been dramatically improved from the initial 3.8% to recently certified value of 25.2% [1–24]. However, there exist unavoidable shortcomings in these Pb-based high-efficiency PSCs (e.g., MAPbI_3_, FAPbI_3_, Cs_0.05_FA_0.85_MA_0.10_Pb(I_0.97_Br_0.03_)_3_), that is, the element lead is toxic to the environment and organisms and difficult to discharge from the body. Research indicates that the contamination of lead ions to soil and water sources is permanent and generates a very serious negative impact on human, animal, and plant survival [25–34]. The human’s nervous, digestive and blood systems eventually show functional disorders in the case of lead poisoning since lead can enter human’s body, bind with enzyme and finally store in soft tissues and bones such as spleen, kidney, liver, and brain through blood circulation. Generally, most people show lead toxic symptoms when lead intake reaches about 0.5 mg/day [25–27]. Therefore, to guarantee human’s safe and pollution-free natural environment it is necessary to develop some non- or low-toxic metal ions to replace lead as perovskite absorbers of PSCs [28–34].

Generally, metal halide perovskites have a universal chemical formula of ABX_3_, where A is an organic cation, B is a metal cation and X is a halogen anion [35–55]. Here, the organic cation usually includes methylammonium (MA), formamidinium (FA), Cs, or their mixture, and the halogen anion consists of Cl, Br, I or their mixture. For B, previous work has showed that less toxic ions like Sn^2+^, Bi^3+^, Ge^2+^, Sb^3+^, Mn^2+^, and Cu^2+^ could be used as an alternative ion to Pb^2+^ in perovskites to constitute a new lead-free perovskite structure [56–63]. The introduction of these metal cations not only increases the diversity of perovskite species, but also enhances environmentally friendly features of PSCs.

This review mainly focuses on various lead-free perovskite materials and related solar cell applications by summarizing recent work about theoretical bases and experimental studies of those lead-free perovskites. Specifically, it summarizes the replacement of Pb with other possible isovalent and heterovalent elements proposed recently. Herein, the isovalent elements consisting of Sn (II), Ge (II) and divalent transition metals (e.g., Cu, Fe, Zn), and the heterovalent elements containing Bi(III), Sb (III), Sn (IV), Ti (IV) and double cations of Ag(I)Bi(III) are summarized. The crystal structure and bandgap for all of lead-free materials and their corresponding photovoltaic performance and stability are included. And by comparing various types of lead-free perovskite materials, we finally predict development prospects of lead-free perovskite materials in the future.

## Theoretical Bases of Lead-Free Hybrid Perovskites

First-principles calculation based on density functional theory (DFT) is a modeling method for simulating the electronic structures of many-body systems from atomic-scale quantum mechanics. This method has been used as an important tool for the study of lead-free perovskites [64, 65]. Electronic structures of various lead-free perovskites were calculated by researchers to simulate their electron/hole effective masses, theoretical absorption spectra, carrier mobilities, bandgaps, and other properties related to their potential solar cell applications by first-principles calculations in order to find the optimal MAPbI_3_ substitute [67–70]. Generally speaking, there exist a variety of options of exchange–correlation functions in DFT calculations, which will qualitatively affect the result of DFT calculations. In the past research work, researchers often chose local density approximation (LDA) and generalized gradient approximation (GGA) functionals. Although their functions are relatively simple, the calculation results are generally more reliable. But this does not mean that there are no problems. LDA and GGA exhibit serendipitous cancellations between their exchange–correlation parts, which lead to unphysical electron self-interactions. This result further causes the local (LDA) and semilocal (GGA) functions to essentially underestimate the bandgaps of solids by 30–100% [67, 68]. To solve such problems, methods/functions including the many-body perturbation (GW) approximation [65], time-dependent DFT (TDDFT) [66], PBE0, HSE03, and HSE06 hybrid functionals [64–70], and more recent delta self-consistent-field method have been developed and applied to recent theoretical calculations of lead-free perovskites. It is worth emphasizing that in the presence of heavy atoms, spin–orbit coupling (SOC) must be included in the calculation to achieve a more accurate prediction of the electronic structure.

**Fig. 1:**
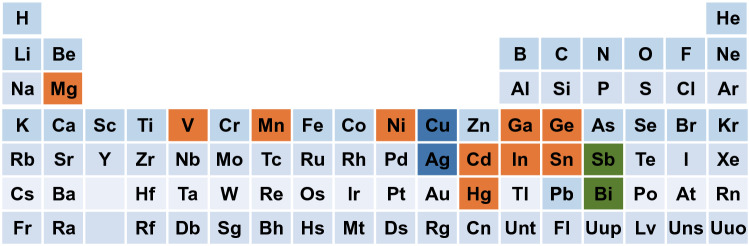
Potential elements to substitute Pb. The orange shading on the periodic table marks the screened elements by Filip et al. that can replace Pb. The green shading of the VA group heterovalent elements and the blue shading of transition metal elements have also been calculated or proved to substitute Pb. (Color figure online)

So far, researchers have already had a certain understanding of the lead-free perovskite system after several years of targeted research. The core of research on lead-free perovskite materials is the replacement of Pb element. Researchers have screened out a series of elements that could replace Pb through theoretical calculations (Fig. 1).

Filip et al. considered stability and desired bandgap as two concurrent prerequisites for suitable candidates of lead-free perovskites [66]. They used randomly displaced structures (atom positions and lattice parameters) to investigate the stability of possible lead-free perovskites. Making the stability and desired calculated bandgap as two concurrent conditions, the crystal structure retains the perovskite geometry after relaxation of the shaken configurations, and the relativistic bandgap is smaller than 2.0 eV. Finally, they identified 10 compounds out of 248 candidates for possible solar cell applications with high-throughput screening, as shown in Fig. 1. After calculation, AMgI_3_ exhibited as a promising candidate with tunable bandgap between 0.9 and 1.7 eV (1.7 eV for CsMgI_3_, 1.5 eV for CH_3_NH_3_MgI_3_ and 0.9 eV for CH(NH_2_)_2_MgI_3_) and low electron effective mass, which is caused by exceptionally dispersive conduction bands [66]. Therefore, Filip et al. deduced that the substitution of Pb by Mg was theoretically feasible to reduce the toxicity of metal halide perovskites without losing solar cell efficiency substantially. In subsequent research work, the researchers have also proved that Cu, Ag, Bi and Sb can also replace Pb to form perovskites (Fig. 1) [62–66].

**Fig. 2:**
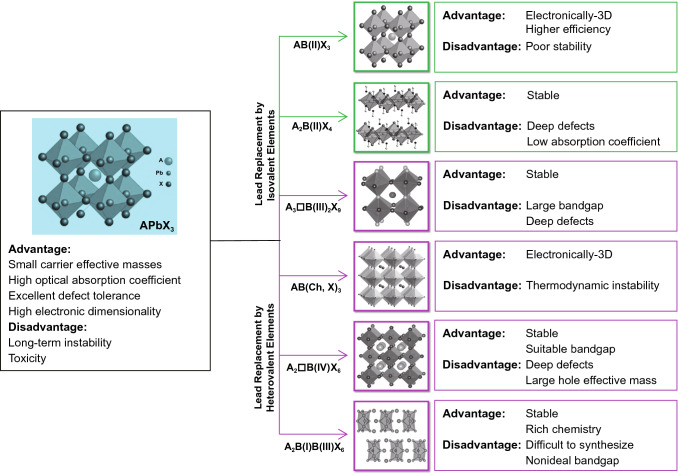
Schematic illustration of the approaches and consequences of potential Pb replacement

In addition, it is possible to use valence state replacement to select suitable non-toxic elements by using the first-principles calculation [59, 64–75]. Usually, as shown in Fig. 2, we can replace the Pb element with either homovalent elements such as Sn, Ge, and Cu, or heterovalent elements such as Sb and Bi. To maintain the charge neutrality, the heterovalent replacement can be divided into three subcategories: cation splitting, mixed valence anion and ordered vacancy. Cation splitting is to form a double perovskite structure with a chemical formula of A_2_B(I)B(III)X_3_ by splitting Pb into a combination of monovalent and trivalent cations. Introducing mixed valence anions is the case with a single valence cation but two valence anions, whose general chemical formula is AB(Ch, X)_3_, where Ch represents a chalcogen element and X represents a halogen element. The perovskite can maintain electrical neutrality via forming ordered vacancies. Such substitutions can also be divided into two types: the B(III) compounds with a formula of A_3_□B(III)X_9_ and the B(IV) compounds with a formula of A_2_□B(IV)X_6_. Here, the sign of □ indicates vacancies. However, the formation of vacancies cuts the original 3D perovskite structure into a low dimension crystal structure, thereby reducing the electronic dimension and then affecting the optoelectronic performance [67].

Researchers have conducted a lot of theoretical studies on the Pb replacement with various elements, e.g., Sn, Ge, and Bi [57–62]. These theoretical studies reveal the advantages and disadvantages of different lead-free perovskite materials. In the substitution of homovalent elements, take Sn-based perovskite MASnI_3_ as an example. In the process of studying the optoelectronic properties of MASnI_3_ using the GW + SOC method, the researchers found that compared with MAPbI_3_, MASnI_3_ showed a stronger *s*-*p* antibond coupling near the maximum value of the valence band (VBM) [68]. This phenomenon is due to the fact that the Sn 5s lone pair is shallower and more active than the Pb 6s lone pair. In addition, because of much weaker SOC, the Sn 5p orbital is shallower and less dispersive than the Pb 6p orbitals. As a result, compared to MAPbI_3_, the bandgap of MASnI_3_ is reduced. Unfortunately, defect calculations indicated that the high-energy Sn 5s^2^ state makes the Sn-I bond easy to break, forming high-density Sn vacancies, resulting in an excessively high defect density (> 10^17^ cm^−3^) in the Sn-based perovskite [68]. Similar to Sn-based perovskite, theoretical calculations by Ming et al. indicated that the Ge vacancy in Ge-based perovskite is the dominant defect with shallow transition levels and rather low formation enthalpy, which yields high-density holes, particularly at Ge-poor synthesis conditions [33]. At the same time, Sun et al. pointed out that the stability of MAGeI_3_ is better than that of MASnI_3_ based on the calculation of the formation energy of the compound. In addition, they calculated the electronic structure and improved bandgap results through the hybrid function HSE06 and the spin–orbit coupling [36]. The calculated band structure showed that the bottom of the conduction band and the top of the valence band are very dispersed, which corresponds to low electron/hole effective masses. These results mean that the replacement of Pb in perovskite by Ge is possible.

In the theoretical study of heterovalent replacement, using Cs_3_Bi_2_I_9_ as an example, the calculations using hybrid Heyd–Scuseria–Ernzerhof (HSE) functional with SOC indicated that Cs_3_Bi_2_I_9_ exhibits an indirect bandgap of 2.10 eV from the Γ point to the K point [40, 51]. Moreover, the defect calculations by Ghosh et al. indicated that most of the defects in Cs_3_Bi_2_I_9_ that have low formation energies generate deep level states in the bandgap, which act as recombination centers [51]. Fortunately, this type of perovskite material has been calculated to own excellent thermodynamic stability.

In addition, the chalcogen–halogen hybrid perovskite AB(Ch, X)_3_ was systematically studied by Sun et al. with Ab initio molecular dynamics, GGA functions and DFT-D3 van der Waals scheme [52]. According to relevant calculations, they predicted that MABiSI_2_ and MABiSeI_2_ have the best bandgap (1.3–1.4 eV) for solar energy collection materials. However, in later studies, Hong et al. combined DFT calculations and solid-state reactions to prove that all proposed AB(Ch, X)_3_ perovskites are thermodynamically unstable [34]. Although this result is disappointing, it also reminds us that stability analysis is equally important in the theoretical calculation of the discovery of new lead-free perovskites.

Recently, the theoretical calculation has proved the 0D electronic dimension and larger bandgap of double perovskite A_2_B(I)B(III)X_6_ with the use of alkali metal B(I) cation [28, 29]. Actually, transition metal B(III) cations with multiple oxidation states and/or partially occupying d or f orbitals are not ideal for the design of photovoltaic absorbers because they may introduce deeper defect states and localized bandedges. Finally, regarding the screening of X anions, it is known that iodides and bromides have the potential of obtaining narrower bandgaps, but they are thermodynamically unstable, while fluorides and chlorides are relatively stable but usually have larger bandgaps [75–78]. The above theoretical research work undoubtedly played a guiding role in the application of new-type lead-free perovskite materials in perovskite solar cells, resulting in a rapid development of this field.

## Experimental Investigation of Various Types of Lead-Free Hybrid Perovskites

### Lead Replacement by Isovalent Elements

#### Sn-based Perovskite

As a chemical element of Pb’s family (IVA group), Sn^2+^ possesses a homologous lone-pair s orbital and similar radius (1.35 Å) to Pb^2+^ (1.49 Å); therefore, Sn-based perovskites show superior optoelectronic properties similar with Pb [68, 69]. In addition, they also exhibit narrower optical bandgaps (1.2–1.4 eV) and higher carrier mobilities than the Pb-based counterparts. Moreover, due to intrinsic low toxicity of Sn, the Sn-based perovskite absorbers have become the important candidate for lead-free perovskites and gradually turned into the research hotspot in high-performance lead-free PSCs [68–70]. As a result, the Sn-based perovskites are more suitable for efficient single-junction solar cell applications. However, Sn^2+^ is unstable and easily oxidized to Sn^4+^. The formation of Sn^4+^ leads to p-type self-doping in Sn-based perovskites, which will generate numerous Sn vacancies with large background carrier density and rapid recombination of charge carriers [14], leading to serious performance degradation and poor reproducibility of PSCs using Sn-based perovskites as absorbers [26–30].

##### 3D Sn-based Perovskite

1. MASn(I,Br)_3_

**Fig. 3:**
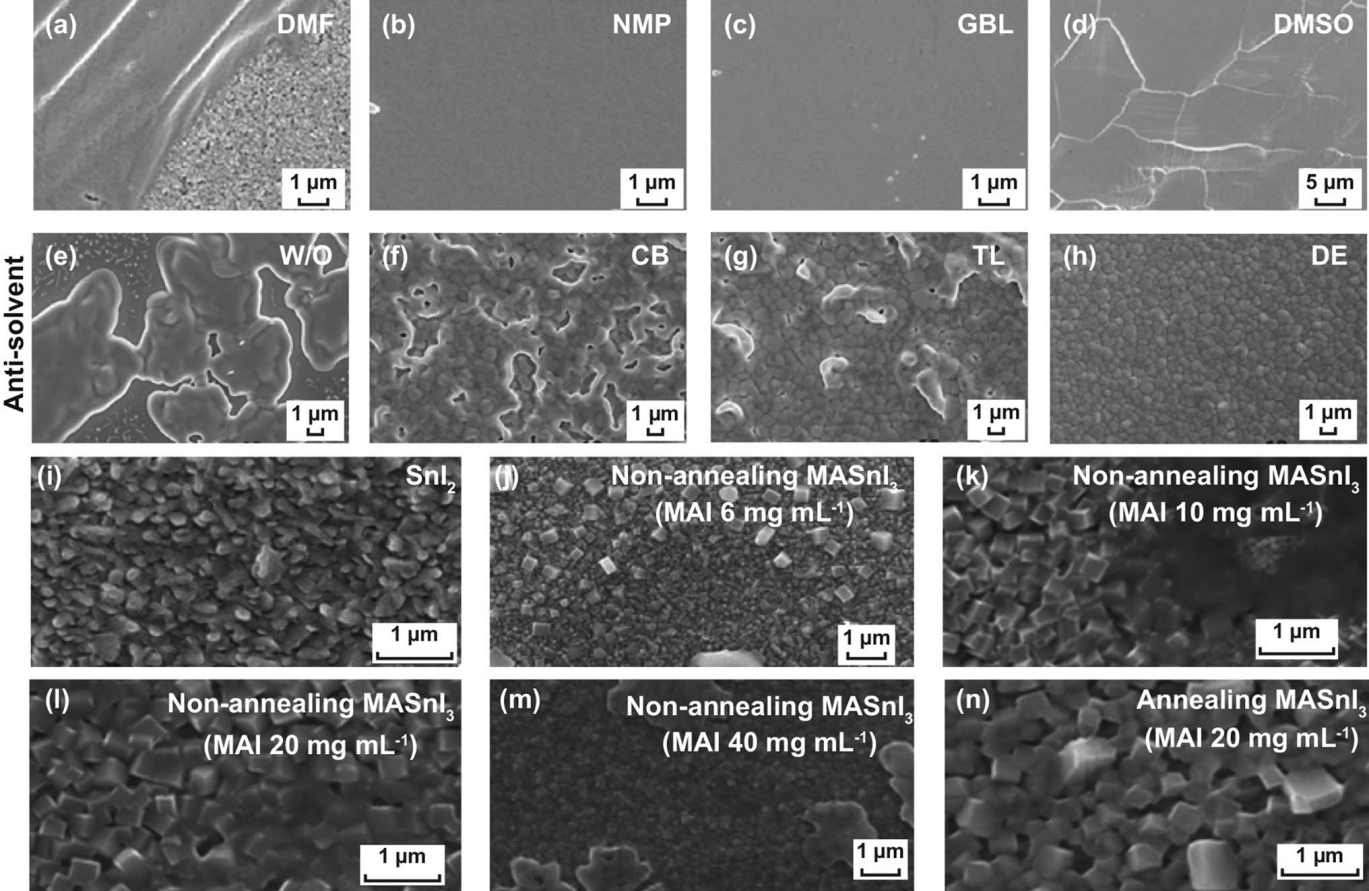
SEM images for MASnI_3_ layer on mesoporous TiO_2_ layer by using **a** DMF, **b** NMP, **c** GBL, and **d** DMSO solvents. Reproduced with permission from Ref. [74]. SEM images for FASnI_3_ films obtained from different antisolvent processes: **e** No dripping, **f** CB, **g** TL and **h** DE. Reproduced with permission from Ref. [96]. SEM images of **i** vapor deposited 100 nm SnI_2_, non-annealing MASnI_3_ films with **j** 6, **k** 10, **l** 20 and **m** 40 mg mL^−1^ MAI precursor solution spin coated and **n** MASnI_3_ films with 20 mg mL^−1^ MAI precursor solution spin coated followed by annealing at 80 °C for 10 min. Reproduced with permission from Ref. [75]

The first MASnIBr_2_ perovskite solar cell was realized with a PCE of 5.7% in 2014 [70], whose PCE was then quickly refreshed to 6.4% by Zhao in the same year [71]. In 2015, Umari et al. investigated the optoelectronic properties of MASnI_3_ with a more advanced many-body perturbation theory involved with SOC [68]. They found MASnI_3_ exhibited a higher charge mobility of 10^2^–10^3^ cm^2^ V^−1^ s^−1^ [68, 69], a smaller direct bandgap of 1.1 eV at the Γ point and a higher absorption coefficient of 1.82 × 10^4^ cm^−1^ in the visible region than MAPbI_3_, whose mobility, bandgap and absorption coefficient of the latter are 10–10^2^ cm^2^ V^−1^ s^−1^, 1.5 eV and 1.80 × 10^4^ cm^−1^, respectively [56, 70]. These pretty results encouraged researchers to pay more attention to Sn-based perovskites. Interestingly, Sn-based perovskite materials exhibit n-type semiconductor characteristics when the internal Sn^2+^ content ratio is high, and p-type semiconductor characteristics when the Sn^4+^ content ratio is high. Until now plenty of efforts have been dedicated to development of high-performance and air-stable Sn-based perovskite solar cells [33, 34]. Poor homogeneity and low coverage are critical issues to be solved in Sn-based perovskites, which usually lead to direct contact between the hole-transporting layer (HTL) and the electron-transporting layer (ETL), thus generating low PCEs [70, 72]. Here, film formation methods suitable for Pb-based perovskites tend to become inefficient for Sn-based cases due to a faster crystallization rate of Sn-based perovskites [73, 74]; for instance, one-step method with common solvent of *N*,*N*-dimethylformamide (DMF) or *γ*-butyrolactone (GBL) hardly realized smooth Sn-based perovskite films on flat substrates. Hao et al. studied the effect of solvent on the crystallization of MASnI_3_ perovskite film and found that adding dimethyl sulfoxide (DMSO) retarded the perovskite crystallization via the formation of a stable intermediate adduct SnI_2_·3DMSO (Fig. 3a), the film formation speed of which was eventually controlled by the evaporation rate of DMSO. As the scanning electron microscopy (SEM) images shown in Fig. 3a–d, the morphologies of MASnI_3_ films with DMSO showed the highest coverage and pinhole–free property on mesoporous TiO_2_ [74]. Moreover, *N*-methyl-2-pyrrolidone (NMP) was also investigated as a solvent to further understand the effect of the intermediate phase on the tin perovskite formation process. As a result, a high-quality, pinhole-free CH_3_NH_3_SnI_3_ films are achieved using NMP as solvents [73, 74].

Multi-step film formation method was another efficient approach to achieve high-quality Sn-based perovskite films [19, 56, 75, 76]. Schlettwein et al. reported an approach combined thermal evaporation with solution process to fabricate MASnI_3_ perovskite films [56, 76]. The SnI_2_ layers prepared by vapor thermal deposition could be converted to MASnI_3_ by sequentially spin coating MAI solution. The as-acquired sizes of MASnI_3_ grains were over 200 nm (Fig. 3i–n) [75]. Moreover, the film morphologies were highly dependent on MAI concentrations. With the increasing concentration of MAI, larger and more uniform MASnI_3_ crystals were obtained (Fig. 3i–n). After 10-min thermal annealing, high-quality and dense films were achieved with significantly improved stability. The films remained stable after exposure to air for 90 min under both dark and light conditions.

**Fig. 4:**
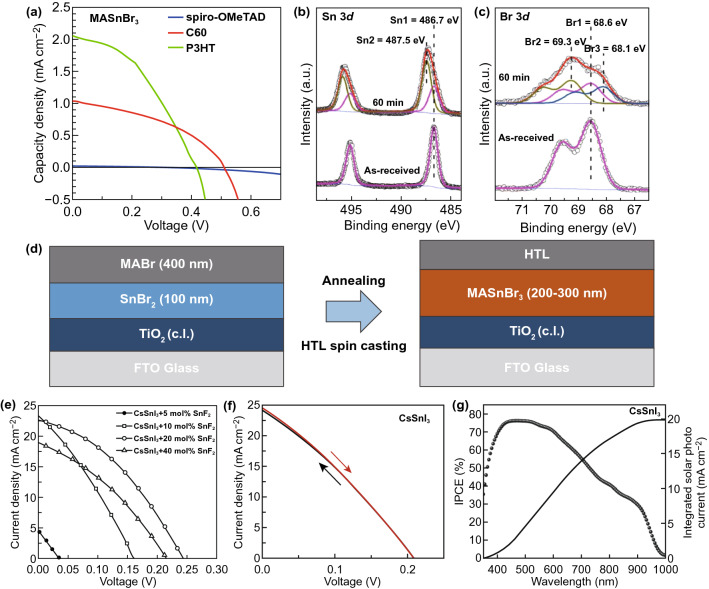
**a** *J*–*V* curves of co-evaporated MASnBr_3_ films with different hole transport materials. **b** Sn 3d and **c** Br 3d XPS results of MASnBr_3_ films as-deposited and after 1 h stored in air. **d** Fabrication of sequential method for MASnBr_3_ films. Reproduced with permission from Ref. [77]. **e** *J*–*V* curves of CsSnI_3_-based PSCs incorporated with different ratios of SnF_2_. **f** *J*–*V* curves of 20 mol % SnF_2_-doped CsSnI_3_ devices showing no hysteresis. **g** IPCE spectrum for 20 mol % SnF_2_-doped CsSnI_3_ devices. Reproduced with permission from Ref. [71]

In addition to the above approaches, Qi et al. invented a method to improve the quality of MASn(I, Br)_3_-based perovskite film. They used co-evaporation and sequential evaporation methods to fabricate MASnBr_3_ perovskite films with SnBr_2_ and MABr as precursors [77]. For co-evaporation, they used a 4:1 MABr/SnBr_2_ deposition ratio (0.4:0.1 Å s^−1^) to simultaneously evaporate the two materials, and finally produced a perovskite film with a thickness of about 400 nm. However, solar cells based on this film only showed a low PCE of 0.35% (Fig. 4a). Such a low PCE was resulted from the oxidation of Sn^2+^ which restrained the generation of excitons, the carriers diffusion, and the final charge extraction. As a melioration, Qi et al. then employed sequential deposition method to decrease oxidation (Fig. 4b, c) and realized a relatively high PCE of 1.12% because oxidation can be significantly avoided by the top MABr layer [77]. The specific operation is first to deposit a 100-nm film of SnBr_2_, and then to precipitate a following 400-nm film of MABr (Fig. 4d). The as-deposited sample was transferred from the vacuum system to a N_2_ glovebox for post-annealing so as to convert the two-layer sample to MASnBr_3_ perovskite. Therefore, sequential evaporation approach could be an efficient approach for fabricating high-quality Sn-based perovskites.

(2) CsSn(I,Br)_3_

In 1974, Fisher et al. first synthesized and characterized all-inorganic CsSnX_3_ compounds [78]. It was not until 2012, Shum et al. reported the application of CsSnI_3_ in perovskite solar cell [79]. A black thin film of CsSnI_3_ with a bandgap of 1.3 eV was fabricated by alternate depositions of SnCl_2_ and CsI on a glass substrate followed by a thermal annealing process. The CsSnI_3_ perovskite solar cell exhibited a PCE of 0.9%. Then in 2014, Mathews et al. reported a CsSnI_3_ PSC incorporated with SnF_2_ to reduce Sn vacancies and obtain a high photocurrent output [71]. The best PCE of 2.02% as well as a short-circuit current density (*J*_sc_) of 22.70 mA cm^−2^, an open-circuit voltage (*V*_oc_) of 0.24 V and a fill factor (FF) of 0.37 was achieved with 20 mol % SnF_2_ (Fig. 4e). Moreover, the devices displayed less hysteresis than those counterparts without SnF_2_ (Fig. 4f). The incident photon-to-current conversion efficiency (IPCE) data clearly indicated that the onset was extended to 950 nm, which corresponded to 1.3 eV of bandgap (Fig. 4g). In 2015, a detailed study on CsSnX_3_ was done by Sabba et al. [80]. Their studies revealed that the CsSnX_3_ materials were semiconductors with a strong tendency to self-doping because the oxidation of Sn^2+^ to Sn^4+^ would generate hole carriers.

It is worth mentioning that CsSnI_3_ as a 3D p-type orthorhombic perovskite possesses a bandgap of 1.3 eV [81], a low exciton binding energy of 18 × 10^−3^ eV [82], and a high optical absorption coefficient of 10^4^ cm^−1^ (comparable to MAPbI_3_) [83]. Therefore, it has the potential to be as a light absorber for lead-free PSCs. The biggest obstacle that hinders rapid development of CsSnI_3_ perovskite solar cell is the instability of CsSnI_3_ and the black phase CsSnI_3_ could be easily converted to yellow phase CsSnI_3_ in atmosphere due to its oxidization [69, 84]. It was reported that the excessive SnI_2_ contributed to the improvements of efficiency and stability of CsSnI_3_-based PSCs [85]. The CsSnI_3_ films with low defect density and high surface coverage were prepared in a Sn-rich environment or at high temperature. The PCE of the device with a configuration of ITO/CuI/CsSnI_3_/fullerene/bathocuproine (BCP)/Al increased from 0.75 to 1.5% in the condition of 10 mol % excessive SnI_2_, and *V*_oc_ and FF remained good within 20 min under light stability. Although *J*_sc_ quickly deteriorated by 10% in the first 50 min, *J*_sc_’s decline rate slowed significantly. The excess of SnI_2_ located at the CsSnI_3_/CuI interface formed an interfacial dipole which acted as a hole-transporting layer from CsSnI_3_ to CuI owing to a favorable vacuum level shift.

After that, some additives have been introduced into precursors to further improve film quality of CsSn(I,Br)_3_ perovskites, which have significantly promoted the performances of CsSn(I,Br)_3_ perovskite solar cells [86–90]. Marshall et al. systematically investigated CsSnI_3_ perovskite films with different Sn halide additives, such as SnF_2_, SnCl_2_, SnI_2_, and SnBr_2_ [90]. The films adding SnCl_2_ additive had the highest pinhole density and the HTL-free devices with 10 mol % SnCl_2_ achieved the highest FF and PCE. Such results were mainly attributed to the formation of ultrathin hole-selective layer of SnCl_2_ at the ITO/CsSnI_3_ interface.

**Fig. 5:**
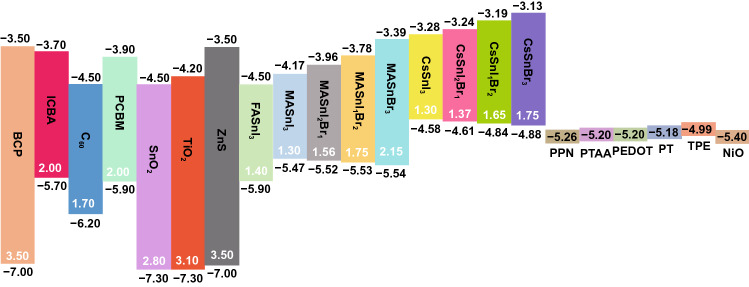
Energy diagram of common Sn-based perovskites, ETLs and HTLs. Unit: eV

On this basis, doping Br into CsSnI_3_ was also proposed. It is noteworthy that the Br-doped CsSnI_3−x_Br_x_ perovskites showed a much higher FF compared to CsSnI_3_ due to the appearance of the negligible overlayer of CsSnI_3−x_Br_x_ [80]. The crystal structure was thus changed from orthorhombic (CsSnI_3_) to cubic (CsSnBr_3_) with the increase in Br component. The onset of optical bandgap edge was also altered from 1.27 eV (CsSnI_3_) to 1.37, 1.65, and 1.75 eV for CsSnI_2_Br, CsSnIBr_2_ and CsSnBr_3_, respectively (Fig. 5). Notably, CsSnI_2_Br, CsSnIBr_2_ and CsSnBr_3_ were suitable for solar cell applications due to their excellent thermal and air stabilities (Table 1) [80].

**Table 1:** Photovoltaic parameters of PSCs based on various Sn-based perovskite absorbers. In the table, c-TiO_2_ and m-TiO_2_ represent compact and mesoporous TiO_2_ layer, respectively

Preparation process	Absorber	Device architecture	*V*_oc_ (V)	*J*_sc_ (mA cm^−2^)	FF (%)	PCE (%)	References
One-step	MASnI_3_	c-TiO_2_/m-TiO_2_/perovskite/spiro-MeOTAD/Au	0.68	16.30	48	5.23	[70]
One-step	MASnI_2_Br	c-TiO_2_/m-TiO_2_/perovskite/spiro-MeOTAD/Au	0.77	14.38	50	5.48	[70]
One-step	MASnIBr_2_	c-TiO_2_/m-TiO_2_/perovskite/spiro-MeOTAD/Au	0.82	12.30	57	5.73	[70]
One-step	MASnBr_3_	c-TiO_2_/m-TiO_2_/perovskite/spiro-MeOTAD/Au	0.88	8.26	59	4.27	[70]
One-step	MASnI_3_	c-TiO_2_/m-TiO_2_/perovskite/spiro-MeOTAD/Au	0.716	15.18	50.07	5.44	[135]
One-step	MASnI_3_	c-TiO_2_/m-TiO_2_/perovskite/spiro-MeOTAD/Au	0.88	16.8	42	6.4	[82]
One-step	MASnI_3_	c-TiO_2_/m-TiO_2_/perovskite/spiro-MeOTAD/Au	0.79	13.40	52	5.49	[124]
One-step	CsSnI_3_ + 20% SnF_2_	c-TiO_2_/m-TiO_2_/perovskite/spiro-MeOTAD/Au	0.201	27.67	29	1.66	[91]
One-step	CsSnI_2_Br + 20% SnF_2_	c-TiO_2_/m-TiO_2_/perovskite/spiro-MeOTAD/Au	0.289	15.06	38	1.67	[91]
One-step	CsSnIBr_2_ + 20% SnF_2_	c-TiO_2_/m-TiO_2_/perovskite/spiro-MeOTAD/Au	0.311	11.57	43	1.56	[91]
One-step	CsSnI_2.9_Br_0.1_ + 20% SnF_2_	c-TiO_2_/m-TiO_2_/perovskite/spiro-MeOTAD/Au	0.222	24.16	33	1.76	[91]
One-step	FASnI_3_ + 20% SnF_2_	c-TiO_2_/m-TiO_2_/perovskite/spiro-MeOTAD/Au	0.238	24.45	36	2.10	[99]
One-step	CsSnBr_3_ + 20% SnF_2_	c-TiO_2_/m-TiO_2_/perovskite/spiro-MeOTAD/Au	0.42	9.1	57	2.17	[89]
One-step	MASnIBr_2_	c-TiO_2_/m-TiO_2_/perovskite/spiro-MeOTAD/Au	0.69	15.9	49	5.36	[134]
One-step	MASnI_3_ + 20% SnF_2_	c-TiO_2_/m-TiO_2_/perovskite/PTAA/Au	0.25	26.1	30	1.94	[137]
One-step	{en}FASnI_3_ + 15% SnF_2_	c-TiO_2_/m-TiO_2_/perovskite/PTAA/Au	0.48	22.54	65.96	7.14	[81]
One-step	{en}FASnI_3_ + 15% SnF_2_	c-TiO_2_/m-TiO_2_/perovskite/PTAA/Au	0.46	22.54	69.74	7.23	[138]
One-step	{PN}FASnI_3_ + 15% SnF_2_	c-TiO_2_/m-TiO_2_/perovskite/TPE/Au	0.44	22.15	60.67	5.85	[109]
One-step	{TN}FASnI_3_ + 15% SnF_2_	c-TiO_2_/m-TiO_2_/perovskite/PTAA/Au	0.40	22.72	61.04	5.53	[109]
One-step	{en}MASnI_3_ + 15% SnF_2_	c-TiO_2_/m-TiO_2_/perovskite/PTAA/Au	0.43	24.28	63.72	6.63	[108]
One-step	MASnBr_3_	c-TiO_2_/m-TiO_2_/perovskite/PTAA/Au	0.307	1.22	36.8	0.14	[139]
One-step	CsSnI_3_ + 20% SnF_2_	c-TiO_2_/m-TiO_2_/perovskite/*m*-MTDATA/Au	0.24	22.70	37	2.02	[88]
One-step	MASnCl_3_	c-TiO_2_/perovskite/CuSCN/Ag	0.576	12.89	55	3.41	[140]
One-step	CsSnI_3_	NiO/perovskite/PCBM/Al	0.52	10.21	62.5	3.31	[141]
One-step	CsSnI_3_	c-TiO_2_/perovskite/spiro-MeOTAD/Au	0.48	8.11	19.8	0.77	[141]
One-step	HEA_0.4_FA_0.6_SnI_3_	c-TiO_2_/m-TiO_2_/perovskite/Al_2_O_3_/C	0.37	18.52	56.2	3.9	[118]
One-step	GA_0.2_FA_0.78_SnI_3_ + 1% EDAI_2_	PEDOT:PSS/perovskite/C_60_/BCP/Ag	0.61	21.2	72	9.6	[119]
One-step	FASnI_3_	PEDOT:PSS/perovskite/PCBM/BCP/Ag	0.49	22.24	65.19	7.15	[101]
One-step	FASnI_3_	SnO_2_/perovskite/PCBM/BCP/Ag	0.55	19.39	68.82	7.34	[102]
One-step	FASnI_3_	PEDOT:PSS/perovskite/C_60_/BCP/Ag	0.63	21.6	74.7	10.17	[103]
One-step	FASnI_3_	PEDOT:PSS/perovskite/C_60_/BCP/Ag	0.628	22.25	74.2	10.37	[104]
One-step	PEA_x_FA_1−x_SnI_3_ + NH_4_SCN	PEDOT:PSS/perovskite/ICBA/BCP/Ag	0.94	17.4	75	12.4	[135]
One-step	FASnI_3_ + 5% PHCl	PEDOT:PSS/perovskite/C_60_/BCP/Ag	0.76	23.5	64	11.4	[136]
Hot-casting	BA_2_MA_3_Sn_4_I_13_ + 100% SnF_2_	c-TiO_2_/m-TiO_2_/perovskite/PTAA/Au	0.229	24.1	45.7	2.53	[120]
Hot-casting	MASnI_3_ + 20% SnF_2_/hydrazine	c-TiO_2_/m-TiO_2_/perovskite/PTAA/Au	0.378	19.92	51.73	3.89	[142]
Hot-casting	CsSnI_3_ + 20% SnF_2_/hydrazine	c-TiO_2_/m-TiO_2_/perovskite/PTAA/Au	0.170	30.75	34.88	1.83	[142]
Hot-casting	CsSnBr_3_ + 20% SnF_2_	c-TiO_2_/m-TiO_2_/perovskite/PTAA/Au	0.367	13.96	59.36	3.04	[142]
Hot-casting	MASnI_3_	PEDOT/perovskite/PCBM/Al	0.595	17.8	29.8	3.2	[143]
Vapor-assisted	MASnI_3_	c-TiO_2_/m-TiO_2_/perovskite/PTAA/Au	0.273	17.36	39.1	1.86	[143]
Vapor-assisted	MASnI_3−x_Br_x_	c-TiO_2_/m-TiO_2_/perovskite/PTAA/Au	0.452	5.02	48.3	1.10	[139]
Solvent-engineering	FASnI_3_ + 10% SnF_2_ + pyrazine	c-TiO_2_/m-TiO_2_/perovskite/spiro-MeOTAD/Au	0.32	23.7	63	4.8	[71]
Solvent-engineering	FASnI_3_ + 20% SnF_2_	c-TiO_2_/m-TiO_2_/perovskite/spiro-MeOTAD/Au	0.38	23.09	60.01	5.27	[97]
Solvent-engineering	MASnI_3_ + 20% SnF_2_	c-TiO_2_/m-TiO_2_/perovskite/spiro-MeOTAD/Au	0.232	26.0	38.6	2.33	[134]
Solvent-engineering	FASnI_3_ + 10% SnF_2_	PEDOT:PSS/perovskite/C_60_/BCP/Ag	0.47	22.07	60.67	6.22	[96]
Solvent-engineering	FASnI_3_ + 20% PEAI + 10% SnF_2_	NiO/perovskite/PCBM/Al	0.59	14.44	69	5.94	[127]
Solvent-engineering	(FA)_0.75_(MA)_0.25_SnI_3_ + 10% SnF_2_	PEDOT:PSS/perovskite/C_60_/BCP/Ag	0.61	21.2	62.7	8.12	[80]
Solvent-engineering	(FA)_0.5_(MA)_0.5_SnI_3_ + 10% SnF_2_	PEDOT:PSS/perovskite/C_60_/BCP/Ag	0.53	21.3	52.4	5.92	[80]
Solvent-engineering	(FA)_0.25_(MA)_0.75_SnI_3_ + 10% SnF_2_	PEDOT:PSS/perovskite/C_60_/BCP/Ag	0.48	20.7	45.2	4.49	[80]
Solvent-engineering	FASnI_3_ + 10% SnF_2_	PEDOT:PSS/perovskite/C_60_/BCP/Ag	0.48	21.3	64.6	6.60	[80]
Solvent-engineering	MASnI_3_ + 10% SnF_2_	PEDOT:PSS/perovskite/C_60_/BCP/Ag	0.46	21.4	42.7	4.29	[80]
Solvent-engineering	0.92FASnI_3_ + 0.08PEAI + 10% SnF_2_	PEDOT:PSS/perovskite/C_60_/BCP/Ag	0.525	24.1	71	9.0	[144]
Solvent-engineering	FASnI_2_Br	PEDOT:PSS/perovskite/C_60_/Ca/Al	0.467	6.82	54	1.72	[89]
Solvent-engineering	MA_0.9_Cs_0.1_SnI_3_	PEDOT:PSS/perovskite/PCBM/Bis-C_60_/Ag	0.20	4.53	36.4	0.33	[114]
Solvent-engineering	FA_0.8_Cs_0.2_SnI_3_	PEDOT:PSS/perovskite/PCBM/Bis-C_60_/Ag	0.24	16.05	35.8	1.38	[114]
Solvent-engineering	FASnI_3_	PEDOT:PSS/perovskite/PCBM/Bis-C_60_/Ag	0.04	11.73	23.4	0.11	[114]
Solvent-engineering	PEA_2_SnI_4_	NiO_x_/perovskite/PCBM/BCP/Ag	0.61	22.0	70.1	9.41	[123]
Solvent-engineering	(BA_0.5_PEA_0.5_)_2_FA_3_Sn_4_I_13_	PEDOT:PSS/perovskite/C_60_/LiF/Al	0.60	21.82	66.73	8.82	[126]
Solvent-engineering	AVA_2_FA_n−1_Sn_n_I_3n+1_	PEDOT:PSS/perovskite/PCBM/BCP/Ag	0.61	21.0	68	8.71	[128]
Solvent-engineering	(4AMP)(FA)_3_Sn_4_I_13_	c-TiO_2_/ZrO_2_/perovskite/C	0.64	14.9	44.3	4.42	[131]
Quantum rods	CsSnI_3_	c-TiO_2_/perovskite/spiro-MeOTAD/Au	0.86	23.2	65	12.96	[145]
Quantum rods	CsSnBr_3_	c-TiO_2_/perovskite/spiro-MeOTAD/Au	0.85	21.23	58	10.46	[145]
Quantum rods	CsSnCl_3_	c-TiO_2_/perovskite/spiro-MeOTAD/Au	0.87	19.82	56	9.66	[145]
Sequential deposition	FASnI_3_ + 10% SnF_2_ + TMA	SnO_2_/C_60_/perovskite/spiro-MeOTAD/Ag	0.31	21.65	64.7	4.34	[83]
Sequential deposition	FASnI_3_ + 10% SnF_2_ + TMA	PEDOT:PSS/perovskite/C_60_/Bis-C_60_/Ag	0.47	22.45	0.68	7.09	[83]
Sequential deposition	FASnI_3_	PEDOT:PSS/perovskite/C_60_/BCP/Ag	0.33	17.78	67.9	3.98	[146]
Thermal evaporation	MASnBr_3_	c-TiO_2_/perovskite/P3HT/Au	0.498	4.27	49.1	1.12	[77]
Thermal evaporation	CsSnBr_3_ + 2.5% SnF_2_	MoO_3_/perovskite/C_60_/BCP/Ag	0.40	2.4	55	0.55	[147]
Thermal evaporation	(PEA, FA) SnI_3_	LiF/PEDOT:PSS/perovskite/C_60_/BCP/Ag	0.47	20.07	74	6.98	[148]
Thermal evaporation	MASnI_3_	PEDOT:PSS/TPD/perovskite/C_60_/BCP/Ag	0.377	12.1	36.6	1.7	[149]
Thermal evaporation	CsSnI_3_	ITO/perovskite/Au/Ti	0.42	4.80	22	0.88	[87]
Direct dropping	MASnIBr_1.8_Cl_0.2_ + 20% SnF_2_	c-TiO_2_/m-TiO_2_/m-Al_2_O_3_/perovskite/C	0.38	13.99	57.3	3.11	[150]
Hot-dropping	CsSnIBr_2_ + 60% SnF_2_ + H_3_PO_2_	c-TiO_2_/m-TiO_2_/m-Al_2_O_3_/perovskite/C	0.31	17.4	56	3.2	[151]
Solvent-engineering	FASnI_3_ + PEABr + 10% SnF_2_	PEDOT:PSS/perovskite/C_60_/BCP/Cu	0.45	24.87	63	7.05	[152]
Solvent-engineering	FASnI_3_ + 2.5% N_2_H_5_Cl + 10% SnF_2_	PEDOT:PSS/perovskite/PCBM/BCP/Ag	0.455	17.64	67.3	5.40	[153]
Solvent-engineering	FASnI_3_ + 12% SnF_2_	PEDOT:PSS(PEG)/perovskite/PCBM/BCP/Ag	0.455	17.64	67.3	5.40	[153]
Solvent-engineering	MASnI_3_ + 20% SnF_2_	PEDOT:PSS/perovskite/C_60_/BCP/Ag	0.45	11.82	40	2.14	[154]
Solvent-engineering	FASnI_3_ + 1% EDAI_2_	PEDOT:PSS/perovskite/C_60_/BCP/Ag	0.583	21.3	0.72	8.9	[155]
Solvent-engineering	FASnI_3_	PEDOT:PSS/perovskite/C_60_/BCP/Ag	0.638	21.95	0.725	10.16	[156]

(3) FASn(I,Br)_3_

Compared to MA^+^ and Cs^+^, FA^+^ is another extensively investigated organic cation with a relatively larger ionic radius. FASnI_3_ is a 3D perovskite with a bandgap of 1.41 eV, which is slightly wider than MASnI_3_ (1.30 eV) and CsSnI_3_ (1.30 eV) [37] but narrower than Pb-based perovskites (~ 1.5 eV) [69]. Moreover, the bandgaps of FASnX_3_ can be tuned with different halides, e.g., 1.68 and 2.4 eV for FASnI_2_Br and FASnBr_3_ [78, 91]. The FASnI_3_ material exhibits a threshold charge-carrier density of 8 × 10^17^ cm^−3^ and a charge-carrier mobility of 22 cm^2^ V^−1^ s^−1^ [92]. In addition, FASnI_3_ has a similar thermal stability but a lower conductivity with respect to MASnI_3_ [93].

Synthesis and characterization of cubic FASnI_3_ perovskite were first investigated in 1997 [94]. Until now, most high-performance Sn-based PSCs are mainly based on FASnI_3_ materials [95–97] because of their better air stability. This is because the trap density of FASnI_3_ (n-type semiconductor) is as low as ~ 10^11^ cm^−3^, preventing water and oxygen from entering into the FASnI_3_ crystal, which has been experimentally proved by Wang et al. [98]. Therefore, the air stability of the FASnI_3_ crystal is higher than MASnI_3_ one.

After the first successful incorporation of SnF_2_ into CsSnI_3_, Mathews group further verified similar effect of SnF_2_ additive on performance enhancements of FASnI_3_-based solar cells. SnF_2_ additive played a similar role in preventing oxidation of Sn^2+^ to Sn^4+^ and reducing background carrier density, and the FASnI_3_ PSC with 20% SnF_2_ finally achieved a PCE of 2.1% [99].

However, it has been proven that a higher amount of SnF_2_ induced severe phase separation in the FASnI_3_ perovskites films and thus led to performance degradation of solar cells. To solve this issue, Seok et al. introduced pyrazine into DMF/DMSO mixed precursor solution to fabricate high-quality FASnI_3_ films with high coverage and smooth surface [87]. Since the N atoms in pyrazine can accept lone pairs electrons, pyrazine doping can remarkably restrict the phase separation and effectively reduce Sn vacancies [84], with which a dense, smooth, and pinhole-free FASnI_3_ perovskite layer and a pretty good PCE of 4.8% were achieved with high reproducibility. Furthermore, the encapsulated solar cells exhibited good long-term stability, remaining 98% of the initial PCE for over 10-day storage under ambient condition. It is worth mentioning that, in addition to SnF_2_ and pyrazine, hydrazine vapor, hydrazine iodide [69], and Sn powder [100] have also been used as antioxidants in Sn-based perovskites in later work. The application of these antioxidants has a similar effect with SnF_2_, that is, to improve the stability and efficiency of Sn-based PSCs by reducing Sn vacancies and background carriers in Sn perovskites.

In addition, some other special materials have also been served as additives to improve the film quality of perovskites and hinder the oxidation of Sn^2+^. For instance, Chen et al. used the bidentate ligand 8-hydroxyquinoline (8HQ) as an additive in the perovskite [101]. The N and O atoms in the 8HQ can simultaneously coordinate with Sn^2+^ and greatly inhibit the oxidation of Sn^2+^. Meantime, the formation of complex also improved the quality of FASnI_3_ film and reduced defect state-induced non-radiative recombination. Recently, ammonium hypophosphite was introduced into the FASnI_3_ perovskite precursor by Yan et al. to suppress the oxidation of Sn^2+^ and assist the growth of perovskite grains, resulting in improved perovskite film quality and reduced defect density, and the final device showed a PCE of 7.3% [102]. More importantly, about 50% of their original PCE was maintained after the 500 h storage in air. Soon after, Han et al. employed a π-conjugated Lewis base molecule 2-cyano-3-[5-[4-(diphenylamino)phenyl]-2-thienyl]-propenoic acid (CDTA) with high electron density to systematically control the crystallization rate of FASnI_3_ perovskite [103]. They obtained a compact and uniform perovskite film with greatly increased carrier lifetime via forming stable intermediate phase with the Sn-I frameworks. Meanwhile, the introduction of the π-conjugated system also retarded the permeation of moisture into perovskite crystal, which significantly suppressed the film degradation in air [103]. The perovskite solar cell prepared on this basis achieved a PCE of 10.1% and maintained over 90% of its initial value after 1000 h light soaking in air. Another work is Han et al. introduced a liquid formic acid as a reducing solvent in the FASnI_3_ perovskite precursor solution to product the FASnI_3_ perovskite film with high crystallinity, low Sn^4+^ content, reduced background doping, and low electronic trap density. As a result, the lead-free tin halide PSC achieved 10.37% efficiency [104].

In addition to additives, the use of antisolvents also greatly promoted the film quality of FASnI_3_ perovskites. Typical antisolvents include chlorobenzene (CB) [105], toluene (TL) [106], diethyl ether (DE) [107], and other aprotic nonpolar solvents. Here, it is noteworthy that the antisolvents should be miscible with DMSO solvent, but insoluble for perovskites. Liao et al. used such a solvent-engineering method to fabricate FASnI_3_ perovskites and tuned the film morphologies with different antisolvents (Fig. 3e–h) [96]. They obtained high-quality, uniform and fully covered FASnI_3_ perovskite thin films. Among these antisolvents, dripping with diethyl ether onto FASnI_3_ produced the best FASnI_3_ thin films with high uniformity and full coverage. With improved film quality and proper structure design, the champion FASnI_3_ device achieved a PCE of 6.22% with *V*_oc_ of 0.465 V, *J*_sc_ of 22.07 mA cm^−2^, and FF of 60.67%. Furthermore, such device exhibited a weak *J*–*V* hysteresis behavior [96].

Recently, a new type of “hollow” perovskites has been prepared. Chen et al. reported a successful incorporation of medium size cations ethylenediammonium (en) into the 3D FASnI_3_ perovskite structure and they denoted it as {en}FASnI_3_ [91, 108]. Typically, change of A-site cation only results in a small bandgap alteration due to structural distortion, but for {en}FASnI_3_, its bandgap can be tuned in a wide range of 1.3–1.9 eV via simply increasing the en amount [108]. Actually, adding en could improve film morphology, reduce background carrier density and increase carrier lifetime, all of which contribute to the resultant PCE and stability of Sn-based solar cells [70]. Based on this viewpoint, the {en}FASnI_3_ device achieved a 7.14% PCE with higher *V*_oc_ and FF. Similar results were also observed in MASnI_3_ and CsSnI_3_ solar cells [108]. Recently, Kanatzidis et al. also reported two other diammonium cations of propylenediammonium (PN) and trimethylenediammonium (TN) forming new hollow perovskites of {TN}FASnI_3_ and {PN}FASnI_3_ [109]. TN and PN with slightly larger size than en also improved device performances. The FASnI_3_ absorbers mixed with 10% PN and 10% TN achieved enhanced PCEs of 5.85% and 5.53%, respectively [109].

(4) Mixed A Cations Sn-Based Perovskites

**Fig. 6:**
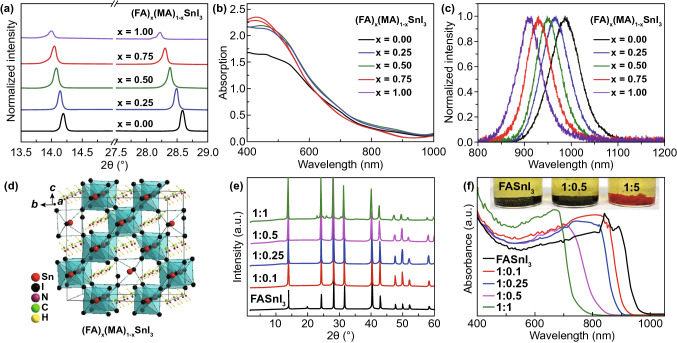
**a** XRD patterns of one-step deposited (FA)_x_(MA)_1−x_SnI_3_ (x = 0.00, 0.25, 0.50, 0.75, and 1.00) films on ITO/poly(3,4-ethylenedioxythiophene)-poly(styrenesulfonate) (PEDOT:PSS) substrates. **b** Absorption spectra and **c** normalized PL spectra of the different perovskite films on quartz substrates. **d** A 2 × 2 × 2 supercell of (FA)_2_Sn_2_I_6_ depicting a model of the hollow perovskite with two SnI_2_ vacancies [(FA)_16_Sn_14_I_44_]. **e** XRD patterns and **f** optical absorption of the {en}FASnI_3_ perovskite crystals with various molar ratios of FA to {en}. Reproduced with permission from Ref. [88]

Metal halide perovskites with mixed cations have been widely used in Pb-based PSCs, part of which achieved record efficiencies and good stability due to cation mixture-triggered film morphology improvement and water and oxygen resistance increase as well as inhibition of carrier recombination within devices [20, 22, 110–113]. In this case, it is also a powerful strategy to develop highly efficient, stable Sn-based PSCs with mixed A cations perovskites. Liu et al. first synthesized Sn-based perovskites with mixed cations of MA and Cs and applied MA_0.9_Cs_0.1_SnI_3_ to PSCs [114]. The PCE was low (0.33%) since the device was fabricated without any optimization. Later, Zhao et al. reported 3D Sn-based perovskites with mixed cations of MA and FA (Fig. 6d). Notably, the optical property exhibited an obvious alteration with the ratio of mixed cations (Fig. 6a–c, e–f). Among them, (FA)_0.75_(MA)_0.25_SnI_3_ perovskite solar cell with 10 mol % SnF_2_ generated a maximum PCE of 8.12% and an average value of 7.48% ± 0.52% [88].

##### Low-Dimensional Sn-Based Perovskite

**Fig. 7:**
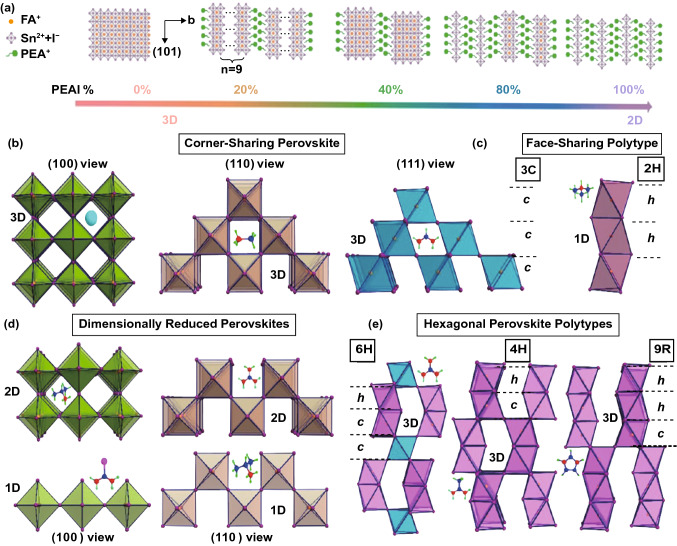
**a** Schematic structures of mixed FA/PEA Sn perovskites with PEAI/FAI doping ratios of 0, 20, 40, 60, 80, and 100%. Reproduced with permission from Ref. [127]. **b** Three alternative views of the archetypal 3D perovskite structure viewed along the (100), (110), and (111) cleavage planes. **c** “Perovskitoid” face-sharing building block of the 1D structures obtained for t > 1 representing the archetypal hexagonal polytype. **d** Perovskite structures obtained through dimensional reduction featuring corner-sharing 2D sheets and 1D chains through the (100) and (110) cleavage planes, respectively. **e** Hexagonal perovskite polytypes obtained from a linear combination of the corner-sharing 3D perovskite along the (111) cleavage plane and the 1D face-sharing polytype. The “h” and “c” symbols indicate hexagonal and cubic layers, respectively, and serve in identifying the layer sequence that characterizes the polytype. Reproduced with permission from Ref. [135]

As an alternative, reduced-dimensional (quasi-2D) perovskites have been widely investigated recently since they have showed superior enduring stability outperforming their 3D counterparts [115–117]. The existence of insulating organic spacer cations, e.g., PN [89], 2-hydroxyethylammonium (HEA) [118], guanidinium (GA) [119], butylammonium (BA) [120], polyethylenimine cation [121], cyclopropylammonium [122], and phenylethylammonium (PEA) on quasi-2D perovskites, restrains self-doping effect and suppresses ion migration [115, 121], thus contributing to ameliorated moisture, oxygen, and thermal stability. However, the emergence of these insulating long-chain organic cations also leads to the anisotropy characteristics of the crystal, which significantly reduces device performance. That means the resulting quasi-2D PSCs with enhanced stability are at the expense of declining performance due to blocked charge transportation of these insulating organic spacers along vertical direction [95, 120, 123]. The highly vertically oriented perovskite films are considered as a prerequisite to address the low-efficiency issue in quasi-2D PSCs since in this situation these inorganic slabs are aligned in perpendicular to the substrates [124, 125]. Various methods of forming highly vertically oriented films have been developed in Pb-based quasi-2D perovskites, but unfortunately, most of which are unsuitable for Sn-based counterparts due to different properties between Pb- and Sn-based perovskites [123, 124]. In the published cases of Sn-based PSCs, Kanatzidis et al. reported 2D Ruddlesden–Popper (RP) perovskites (CH_3_(CH_2_)_3_NH_3_)_2_(CH_3_NH_3_)_n−1_Sn_n_I_3n+1_ with perpendicular orientation by using *N,N*-dimethylformamide as solvent [120]. For *n* = 4, perpendicularly oriented 2D perovskites were fabricated whether the substrates were heated or not, while for *n* = 3, only hot substrates of 120 centigrade were able to intensify perpendicular growth of 2D films. Huang et al. introduced mixed *n*-butylamine and PEA organic cations into 2D RP Sn perovskite to control the crystallization process and formed highly vertically oriented [(BA_0.5_PEA_0.5_)_2_FA_3_Sn_4_I_13_] 2D RP perovskites [126]. Benefitting from it, the PCE of the 2D Sn-based PSC was improved to 8.82%. Liao et al. reported low-dimensional Sn-based perovskites by using FA and PEA mixed cations (Fig. 7a), and they adjusted the orientation of the perovskite domains by altering the PEA/FA ratios [127]. A highly oriented perovskite film perpendicular to the substrate was realized by adding 20% PEA. As a result, the FASnI_3_ perovskite solar cells with 20% PEA achieved a maximum PCE of 5.94% with enhanced stability [127]. Recently, Yuan et al. used the bifunctional cation 5-ammoniumvaleric acid (5-AVA) as the spacer to fabricate AVA_2_FA_*n*−1_Sn_*n*_I_3*n*+1_ (〈*n*〉 = 5) quasi-2D Sn-based perovskite, and by introducing appropriate amount of ammonium chloride (NH_4_Cl) additive, they obtained highly vertically oriented quasi-2D perovskite films which eventually enhanced the transportation of charge carriers between electrodes [128]. Actually, the Cl doping can also improve the electrical conductivity of perovskite film, as demonstrated by Li et al. [129].

In recent work, Padture et al. first reported the synthesis and photovoltaic performance of low-dimensional Dion–Jacobson Sn(II)-based halide perovskites (4AMP)(FA)_n−1_Sn_n_I_3n+1_, but the PSC based on this perovskite only achieved an efficiency of 2.15% [130]. Compared with the Sn-based perovskite of the RP layered with a van der Waals gap between the adjacent unit cells, the unique structure of DJ layered Sn-based perovskites allows the inorganic stacks to be more uniform and closer, rendering better alignment and less displacement of perovskites [131]. Moreover, the shortened interlayer distance might reduce the barrier of charge transfer, which will benefit the charge transport of derived quasi-2D perovskite devices. It is particularly worth mentioning that the growth of the DJ phase perovskite layer can be controlled by selecting different diammonium organic cations as the intermediate spacer layer so that the growth direction is perpendicular to the substrate, which facilitates the transfer and collection of charge through the electrode [124, 132]. These characteristics will make DJ Sn-based perovskites to be a promising class of materials for photovoltaic applications.

Kanatzidis et al. reported some hexagonal polytypes and low-dimensional structures of hybrid Sn iodide perovskites, which employed various small cations such as trimethylammonium (TMA^+^), imidazolium (IM^+^), guanidinium (GA^+^), ethylammonium (EA^+^), acetamidinium (ACA^+^), and isopropylamine (IPA^+^) (Fig. 7b–e) [133]. Due to the moderate size of these cations, a variety of new ASnX_3_ polytypes were formed by combinations of corner-sharing octahedra (perovskite) and face-sharing octahedra (perovskitoid), which have the potential for lead-free solar cells [134]. Finally, we summarize the current research results of Sn-based perovskite solar cells in Table 1 [70, 71, 80–83, 87–89, 96, 97, 101–104, 118–120, 124, 133–156].

To further improve the efficiency of tin-based perovskite, device structure manipulation was also conducted by Ning et al., who used indene-C_60_ bisadduct as the ETL to fabricate solar cells based on PEA_x_FA_1−x_SnI_3_ perovskite, yielding a high efficiency of 12.4% [135]. In addition to efficiency, the stability issue of tin-based PSCs also restrained their applications. Mathews et al. encapsulated FASnI_3_ PSCs and realized more stable PSCs with over 80% of its initial PCE being maintained after 1-month storage in a N_2_ environment, which is much better than the unencapsulated counterparts that fully decomposed within two weeks [71]. In addition, Kanatzidis et al. studied the encapsulated 2D (BA)_2_(MA)_3_Sn4I_13_ PSCs and their test results showed that the encapsulated device retained more than 90% of its initial performance after 1 month and dropped only to ~ 50% after 4 months in a glove box full of N_2_ [120]. This encouraging stability is closely related to the intrinsically stable 2D perovskite structure as well as the encapsulation that sufficiently blocked the invasion of water and oxygen. Other researchers have also proposed in their respective work that it is possible to package Sn-based solar cells to improve stability [71, 103, 104, 120, 136, 155, 156].

In summary, there is still a large room for efficiency and stability enhancements of Sn-based perovskites. Future works should focus on optimization of perovskite composition and selection of effective additives to fabricate stable and high-quality perovskite film with less Sn^2+^ oxidation. Besides, reasonable choice of hole/electron transport materials is very critical for enhancing device performance and stability of the Sn-based PSCs. Protecting of solar cells with industrial encapsulation techniques will also be a viable approach to improve the long-term stability of the Sn-based PSCs and push it to the commercial market.

#### Ge-based Perovskite

Previous reports have shown that metal ions owning external *ns*^2^ electronic structure with low ionization energy can enhance light absorption efficiency and carrier diffusion length of ABX_3_ structure. Ge^2+^ has similar outer ns^2^ electronic structure (4s^2^) to Sn^2+^ (5s^2^) and Pb^2+^ (6s^2^), but smaller ionic radii than Sn^2+^ and Pb^2+^ [64]. Ge-based perovskites containing MAGeI_3_, CsGeI_3_ and FAGeI_3_ have also been demonstrated to be stable at temperature of up to 150 °C. According to these data, Mathews et al. considered that Ge could replace Pb as a new type of lead-free perovskite material [64]. Actually, the bandgaps of the Ge-based perovskites can be adjusted close to the conventional lead halide perovskite of 1.5 eV via altering A-site cations.

**Fig. 8:**
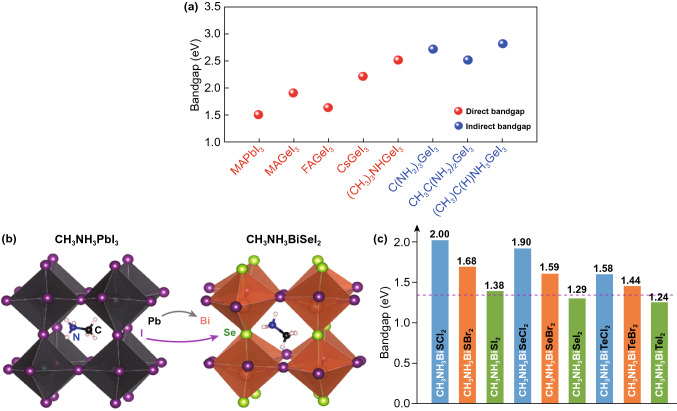
**a** Bandgaps of various Ge-based perovskite materials compared with MAPbI_3_. **b** Atomic structures of MAPbI_3_ and MABiSeI_2_. **c** Calculated bandgaps of CH_3_NH_3_BiXY_2_ compounds (with X = S, Se, Te and Y = Cl, Br, or I) using the Heyd–Scuseria–Ernzerhof functional with spin–orbit coupling. The dashed line marks the optimal bandgap for single-junction solar cell according to the Shockley–Queisser theory. Reproduced with permission from Ref. [52]

Kanatzidis et al. synthesized a series of AGeI_3_ perovskite compounds and characterized their structural, electronic and optical properties in 2015 [61]. The bandgaps of the Ge-based perovskites increase with the increased radius of the A cation. For instance, CsGeI_3_ has an optical bandgap of 1.63 eV, whereas other AGeI_3_ perovskites universally exhibit wide bandgap values of ≥ 2.0 eV. These AGeI_3_ perovskites are rhombohedral crystal structures at room temperature [60]. Kanatzidis et al. [61] used the pyramidal [GeI_3_]^−^ building block to synthesize a series of Ge-based perovskites and adjusted their A-site cations. The experimental results showed that CsGeI_3_, MAGeI_3_, FAGeI_3_ and CH_3_C(NH_2_)_2_GeI_3_ were 3D perovskite structures with direct bandgaps of 1.6, 1.9, 2.2, and 2.5 eV, respectively, while C(NH_2_)_3_GeI_3_, (CH_3_)_2_C(H)NH_3_GeI_3_, and (CH_3_)_3_NHGeI_3_ were 1D infinite chain structures separately with indirect bandgaps of 2.7, 2.5, and 2.8 eV, among which CsGeI_3_ exhibited the highest optical absorption coefficient (Fig. 8a) and showed much more application potential in photovoltaic field.

**Fig. 9:**
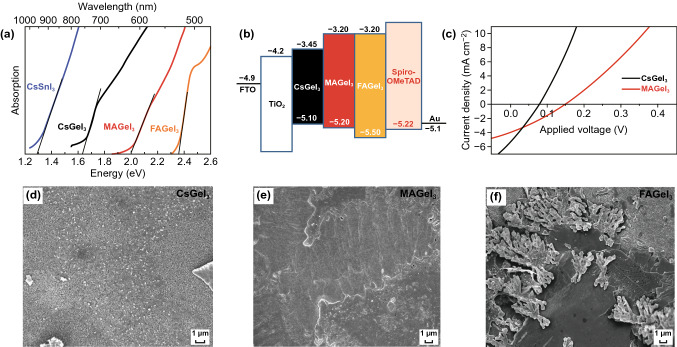
**a** Tauc plot for CsSnI_3_, CsGeI_3_, MAGeI_3_, and FAGeI_3_ showing optical bandgaps of 1.29, 1.63, 2.0, and 2.35 eV, respectively. **b** Energy level pattern for the PSCs with CsGeI_3_, MAGeI_3_, and FAGeI_3_ absorbers. **c** Comparison of *J*–*V* curves of CsGeI_3_ and MAGeI_3_ solar cells. **d-f** SEM images for CsGeI_3_, MAGeI_3_, and FAGeI_3_ films both deposited on the compact TiO_2_/mesoporous TiO_2_ substrates. Reproduced with permission from Ref. [60]

The photoluminescence (PL) measurements of CsGeI_3_ single crystals exhibited two peaks at 0.82 μm (1.51 eV) and 1.15 μm (1.08 eV), which were respectively assigned to the interband transition at α. k = (π/a) (111) and the energy band change (Fig. 9a). The energy bands of the Ge-based perovskites with different A cations are shown in Fig. 9b. Fourier transform infrared spectroscopy showed that the transparent range of CsGeI_3_ can be extended from ~ 2 to > 12 μm. The short-wave cutoff is mainly limited by the energy band, and the longest infrared transparency wavelength may originate from the phonon absorption of crystal lattice, and a specific rhombohedral crystal structure can be generated at room temperature [157]. Mathews et al. used hypophosphorous acid to improve the solubility of Ge perovskite precursor in organic solvent and prepared Ge-based perovskite films [60]. According to the SEM images (Fig. 9d–f), the films of MAGeI_3_ and CsGeI_3_ exhibited nearly full-coverage morphology, but the FAGeI_3_ film showed a poor quality. Based on these films, they then fabricated devices on mesoporous TiO_2_ structures. MAGeI_3_ and CsGeI_3_ solar cells showed *J*_sc_s of 4.0 and 5.7 mA cm^−2^ and PCEs of 0.11 and 0.2%, respectively, as shown in Fig. 9c. In contrast, FAGeI_3_ solar cell with poor film morphology displayed no photocurrent.

Kopacic et al. [158] demonstrated that chemical composition engineering of MAGeI_3_ significantly improved performances of solar cells and simultaneously enhanced their stability. By introducing bromide anion to partially replace idiode, the PCE based on MAGeI_2.7_Br_0.3_ was increased up to 0.57%.

Generally, Ge-based perovskites exhibit obviously different energy structures from the Pb-based ones and thus suitable ETL and HTL should be optimized to improve charge extraction efficiency in the device. More importantly, stabilization of Ge^2+^ in Ge-based perovskite is also a pretty challenging task to be solved. Otherwise, the stability issue and extremely low PCE will limit broader photovoltaic applications of this kind of perovskites.

#### Transition Metal Halide Perovskite

Divalent transition metal cations (e.g., Cu^2+^, Fe^2+^, Zn^2+^) have also been considered as the candidates of Pb-free perovskites, which can be synthesized via a variety of routes to tune photovoltaic properties [63, 159]. Because of small ionic radii for transition metal ions (e.g., 0.73 nm for Cu(II) and 0.78 nm for Fe(II)) that far deviated from tolerance factor of 1, 3D structure (e.g., K_2_NiF_4_) cannot be maintained and it turns into stable 2D layered structure along < 100 > , < 110 > and < 111 > orientations [160].

**Fig. 10:**
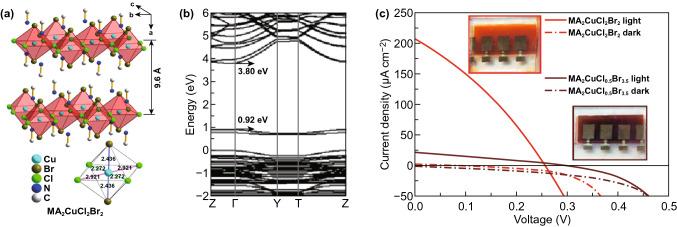
**a** Crystal structure of MA_2_CuCl_2_Br_2_, showing the alternation of organic and inorganic layers and the Cu-X bond lengths in the inorganic framework. **b** Electronic band structure of MA_2_CuClBr_3_ investigated by DFT simulation. **c** *J*–*V* curve of solar cells sensitized with MA_2_CuCl_2_Br_2_ (red) and MA_2_CuCl_0.5_Br_3.5_ (brown) under 1 sun of light illumination. The red and brown dashed lines represent dark current. Reproduced with permission from Ref. [63]

Cu^2+^ is a promising Pb-free perovskite in consideration of its good stability, earth abundance, low-cost and sufficient absorption in near-infrared region. Currently, some Cu-based perovskites have been developed for solar cell applications [63, 161, 162]. Cortecchia et al. first used the 2D crystal structure of MA_2_CuCl_x_Br_4−x_ (Fig. 10a) as a light absorbing layer to fabricate solar cell [63]. They mixed MABr, MACl, CuCl_2_ and CuBr_2_ in DMSO as the precursor solution and tuned the bandgaps of MA_2_CuCl_x_Br_4−x_ from 2.48 to 1.80 eV via changing the Br content (Fig. 10b). Cu-based perovskites exhibited a narrow bandgap with low conduction band compared to other non-transition metal perovskite compounds, whose conduction band is derived from the unoccupied Cu 3d orbitals hybridized with Br/Cl p orbitals. The MA_2_CuCl_x_Br_4−x_ layer was fabricated on the FTO/mesoporous TiO_2_ substrate by spin coating process. A PCE of 0.017% was obtained with extremely low *J*_sc_ of 216 µA cm^−2^ and *V*_oc_ of 0.256 V (Fig. 10c). In addition, they also prepared planar heterojunction cells based on MA_2_CuCl_x_Br_4−x_, but the PCE was far less than 0.017%. The higher PCE for mesoporous structure solar cell arises from improved charge extraction and vertical charge transport along with the destruction of the 2D structure of MA_2_CuCl_x_Br_4−x_ perovskite by mesoporous TiO_2_. The solar cells with MA_2_CuCl_x_Br_4−x_ as the light absorber showed better stability due to the essential role of Cl^−^, but unfortunately, the low absorption coefficient and heavy-mass hole limited their device performances. Subsequent works showed that (C_6_H_5_CH_2_NH_3_)_2_CuBr_4_, (ρ-FC_6_H_5_C_2_H_4_-NH_3_)_2_CuBr_4_ and (CH_3_(CH_2_)_3_NH_3_)_2_CuBr_4_ based solar cells had slightly higher PCEs of 0.2%, 0.51%, and 0.63% [161, 162].

On the whole, Cu-based perovskites are relatively stable compared to other transition metal perovskite materials despite of the universially low conversion efficiency. How to reduce their wide optical bandgap and improve their charge transfer rate is the bottleneck problems limiting the further development of this type of perovskites. We believe that Cu-based perovskites will be an important part of lead-free perovskite solar cells once overcoming these problems.

### Lead Replacement by Heterovalent Elements

#### A_3_B_2_X_9_ Perovskite

**Fig. 11:**
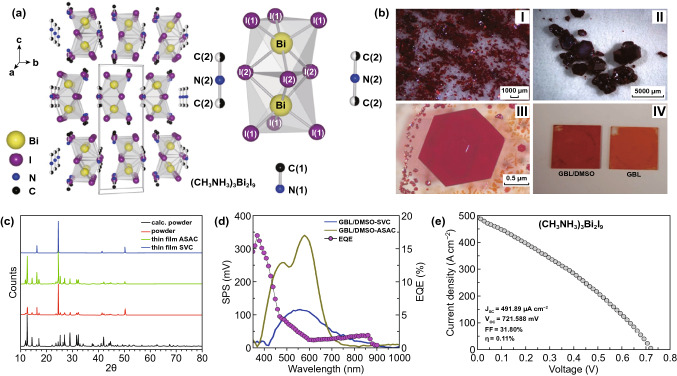
**a** A representation of the layered structure of (CH_3_NH_3_)_3_Bi_2_I_9_, characterized by isolated [Bi_2_I_9_]^3−^ anions and two crystallographic inequivalent CH_3_NH_3_^+^ cations. **b** Small single-crystal powder grown from Bi_2_O_3_ and CH_3_NH_3_I in concentrated HI (I–III). And (CH_3_NH_3_)_3_Bi_2_I_9_ thin films deposited by the antisolvent assisted crystallization method (ASAC) using GBL/DMSO and GBL as solvents and chlorobenzene as antisolvent (IV). **c** Diffraction patterns of (CH_3_NH_3_)_3_Bi_2_I_9_ thin films, powder and simulated powder from single-crystal XRD measurements. **d** EQE of a typical FTO/TiO_2_/(CH_3_NH_3_)_3_Bi_2_I_9_/spiro-MeOTAD/Au device. **e** *J*–*V* scanning of the best-forming device. Reproduced with permission from Ref. [163]

Bi-based perovskites represent a successful example in lead-free perovskite materials family with low toxicity, air stability, and a fair degree of tunability. Abulikemu et al. successfully fabricated (CH_3_NH_3_)_3_Bi_2_I_9_ powders, millimeter-scale single crystals and thin films with solvent-based crystallization methods (Fig. 11a, b) [163]. Compared with Pb-based counterpart, (CH_3_NH_3_)_3_Bi_2_I_9_ perovskite has better stability. In response to the rapid crystallization of perovskites during film formation, the researchers proposed two thin film annealing methods to improve the film-forming quality. One was an antisolvent assisted crystallization route, which dropped antisolvent of chlorobenzene during the spin coating process of precursors and then annealed at 70 °C for 10 min to form perovskite film. The other method was denominated saturated vapor crystallization. The perovskite film was fabricated without dropping chlorobenzene during the spin coating process, and then annealed in a closed Petri dish at 60 °C overnight to slow the solvent evaporation, which generated a highly oriented single-crystal film (Fig. 11b, c). Solar cell devices were fabricated based on these thin films, achieving an efficiency of 0.11%, an FF of 31.80%, an average *J*_sc_ of 491.89 μA cm^−2^ and a *V*_oc_ of 0.7216 V (Fig. 11d, e). However, the external quantum efficiency (EQE) spectrum demonstrated that the device produced photocurrent merely at a short wavelength, with most part of the visible spectrum missed (e.g., above 500 nm) [163]. The explanation given by the researchers was that the depletion of EQE in the (CH_3_NH_3_)_3_Bi_2_I_9_ film was attributed to its inhomogeneity and discontinuity between crystallites in terms of high recombination of photogenerated charges, so the high-quality perovskite film is a prerequisite to achieve a high efficiency.

**Fig. 12:**
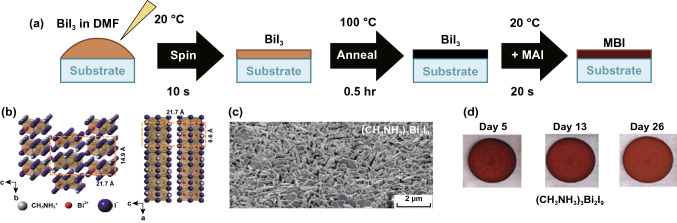
**a** Schematic diagram of (CH_3_NH_3_)_3_Bi_2_I_9_ solution-assisted process. **b** Crystal structure and (001) view of (CH_3_NH_3_)_3_Bi_2_I_9_. **c** SEM image of (CH_3_NH_3_)_3_Bi_2_I_9_ thin film. **d** Stability measurement of (CH_3_NH_3_)_3_Bi_2_I_9_ in air. Reproduced with permission from Ref. [164]

**Fig. 13:**
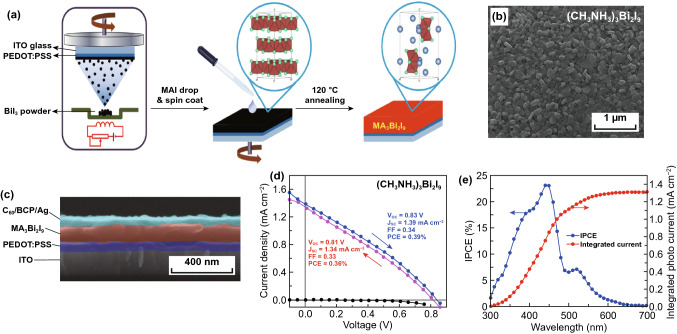
**a** Fabrication procedure and **b** SEM image of (CH_3_NH_3_)_3_Bi_2_I_9_ film. **c** Cross-sectional SEM image of (CH_3_NH_3_)_3_Bi_2_I_9_-based solar cells. **d** Forward and backward scanning and **e** IPCE spectra of the best-forming device. Reproduced with permission from Ref. [166]

Buonassisi et al. used solution-processing and vapor-assisted techniques to synthesize pure phase (CH_3_NH_3_)_3_Bi_2_I_9_ perovskite (Fig. 12a) [164]. They found that the crystal structure of the pure phase (CH_3_NH_3_)_3_Bi_2_I_9_ was composed of alternate MA^+^ and Bi_2_I_9_^3−^ groups (Fig. 13b). The perovskite films prepared by this method were densely packed with high coverage and also showed excellent stability (Fig. 12c, d). The vapor processed films also got a longer PL decay time [164].

Very recently, Zhang et al. made a breakthrough in experimental method of fabricating high-quality (CH_3_NH_3_)_3_Bi_2_I_9_ film [165]. They employed a novel solvent-free high–low vacuum deposition (HLVD) procedure to obtain smooth, compact and pinhole-free polygonal crystal (CH_3_NH_3_)_3_Bi_2_I_9_ film and then fabricated heterojunction solar cells. The HLVD approach proposed here is a two-step alternate vacuum deposition of BiI_3_ and MAI under high- and low-vacuum, which can be further transformed to the resultant (CH_3_NH_3_)_3_Bi_2_I_9_ perovskite. With the approximate 300 nm (CH_3_NH_3_)_3_Bi_2_I_9_ film as the light absorption layer to construct the planar heterojunction PSCs, they not only achieved a high PCE of 1.64% and a high EQE close to 60%, but also obtained a good stability during the measurement of 15 weeks, demonstrating the potential of (CH_3_NH_3_)_3_Bi_2_I_9_ for highly efficient and stable solar cells.

In subsequent research, Ran et al. synthesized a smooth, uniform and pinhole-free (CH_3_NH_3_)_3_Bi_2_I_9_ thin film with a novel two-step evaporation spin coating film fabrication strategy (Fig. 13a, b) [166]. They further optimized film formation conditions by comparing the integrated current from the IPCE measurements and formed compact (CH_3_NH_3_)_3_Bi_2_I_9_ thin films. By taking advantage of the optimized compact thin film, they manufactured inverted planar heterojunction photovoltaic device with a PCE of 0.39% and a high *V*_oc_ of 0.83 V, which exhibited the lowest loss-in-potential to date in (CH_3_NH_3_)_3_Bi_2_I_9_-based solar cells (Fig. 13c–e). However, there still existed a slight hysteresis effect in the *J*–*V* curves [166]. This work certificates that the film morphology engineering is crucial for improving the optoelectronic performance of Bi-based hybrid perovskites solar cells.

Additionally, Park et al. confirmed more efficient solar cells with MA cations replaced by Cs cations in Bi-based perovskite [59]. On the basis of that research, Johansson et al. introduced a bismuth halide as the light absorber with the chemical composition of Cs_3_Bi_2_I_9_ in their solar cells [167]. The morphology of the Cs_3_Bi_2_I_9_ sample showed hexagonal flakes with size up to 2 μm, which were vertically organized and penetrated into TiO_2_. The TiO_2_ can be seen in the gaps between the large flakes due to the unique perovskite morphology and stacking mode. The best-performing device based on Cs_3_Bi_2_I_9_ showed a *J*_sc_ 2.2 mA cm^−2^, but lower *V*_oc_ and FF values generated a PCE of 0.4% [167].

Although researchers have got some successful examples of Bi-based perovskites, the performances of their perovskite solar cells are still universally low due to the intrinsic large bandgaps, large carrier effective masses, large exciton binding energies, more internal defects and defect intolerance. Design of new Bi-based lead-free perovskite materials avoiding above issues on the basis of theoretical calculation is critical for further development of high-performance solar cells. In addition, accurate control of formation rate with facile process technologies (e.g., moisture assisted growth, vacuum deposition, hot spin coating, and so forth) to improve the film quality of Bi-based perovskite allows for an improved performance of Bi-based lead-free perovskite devices. Here, we summarize the current research results of Bi-based PSCs in Table 2 [59, 161, 163–170].

**Table 2:** Photovoltaic parameters of PSCs based on various Bi-based perovskite absorbers

Preparation process	Absorber	Device architecture	*V*_oc_ (V)	*J*_sc_ (mA cm^−2^)	FF (%)	PCE (%)	References
One-step spin coating	Cs_3_Bi_2_I_9_	c-TiO_2_/m-TiO_2_/perovskite/P3HT/Ag	0.31	0.34	38	0.40	[165]
One-step spin coating	(CH_3_NH_3_)_3_Bi_2_I_9_	c-TiO_2_/perovskite/spiro/Au	0.72	0.49	31.8	0.11	[161]
One-step spin coating	(CH_3_NH_3_)_3_Bi_2_I_9_	c-TiO_2_/m-TiO_2_/perovskite/spiro-MeOTAD/Au	0.68	0.38	88	0.22	[166]
One-step spin coating	(CH_3_NH_3_)_3_Bi_2_I_9_	c-TiO_2_/m-TiO_2_/perovskite/spiro-MeOTAD/Ag	0.68	0.52	33	0.12	[59]
One-step spin coating	Cs_3_Bi_2_I_9_	c-TiO_2_/m-TiO_2_/perovskite/spiro-MeOTAD/Ag	0.85	2.15	60	1.09	[59]
One-step spin coating	(CH_3_NH_3_)_3_Bi_2_I_9_Cl_x_	c-TiO_2_/m-TiO_2_/perovskite/spiro-MeOTAD/Ag	0.04	0.18	38	0.03	[59]
One-step spin coating	(CH_3_NH_3_)_3_Bi_2_I_9_	c-TiO_2_/m-TiO_2_/perovskite/P3HT/Au	0.35	1.157	46.4	0.19	[167]
One-step spin coating	(CH_3_NH_3_)_3_Bi_2_I_9_	c-TiO_2_/m-TiO_2_/perovskite/PIF8-TAA/Au	0.85	1.22	73	0.71	[168]
Two-step evaporation spin coating	(CH_3_NH_3_)_3_Bi_2_I_9_	PEDOT:PSS/perovskite/C_60_/BCP/Ag	0.83	1.39	37	0.39	[164]
Two-step thermal evaporation	(CH_3_NH_3_)_3_Bi_2_I_9_	c-TiO_2_/m-TiO_2_/perovskite/spiro-MeOTAD/Au	0.83	3.00	79	1.64	[163]
Vapor-assisted solution process	(CH_3_NH_3_)_3_Bi_2_I_9_	c-TiO_2_/m-TiO_2_/perovskite/P3HT/Au	1.01	4.02	78	3.17	[169]
One-step spin coating	Cs_3_Bi_2_I_9_	c-TiO_2_/perovskite/spiro-MeOTAD/Au	0.79	4.45	50.34	1.77	[170]
One-step spin coating	Cs_3_Bi_2_I_9_	c-TiO_2_/perovskite/CuI/Au	0.86	5.78	64.38	3.20	[170]
One-step spin coating	Cs_3_Bi_2_I_9_	c-TiO_2_/perovskite/PTAA/Au	0.83	4.82	57.49	2.30	[170]

Sb is in the same element group as Bi with low toxicity although it is a heavy metal [171, 172]. Halide perovskites based on group-VA cations of Sb^3+^ are promising candidates due to the same lone-pair *ns*^2^ state as Pb^2+^. Through a joint experimental and theoretical study, researchers found that a 0D structure was formed in Sb-based perovskite, which was similar to Bi, but with lower exciton binding energies. Mitzi et al. reported a highly oriented Sb-based perovskite thin film by a two-step deposition approach. Large grain (> 1 μm) and continuous thin films of the lead-free perovskite Cs_3_Sb_2_I_9_ were achieved by annealing the evaporated CsI in SbI_3_ vapor at 300 °C for 10 min, and the 〈111〉-stacked layered perovskite structure was convinced by the X-ray diffraction (XRD) patterns [62]. The layered inorganic Cs_3_Sb_2_I_9_ has a bandgap of 2.05 eV, measured by ultraviolet photoelectron spectroscopy, inverse photoemission spectroscopy and X-ray photoelectron spectroscopy (XPS), which showed similar absorption as high as CH_3_NH_3_PbI_3_. The DFT calculations indicated Cs_3_Sb_2_I_9_ has a nearly direct bandgap in consideration of less than 0.02 eV difference between the direct and indirect bandgaps. Then Mitzi et al. prepared PSCs with the Cs_3_Sb_2_I_9_ material, which produced a low PCE of less than < 1%, a significant hysteresis effect, and an enhanced air stability compared to CH_3_NH_3_PbI_3_ counterpart [62].

**Fig. 14:**
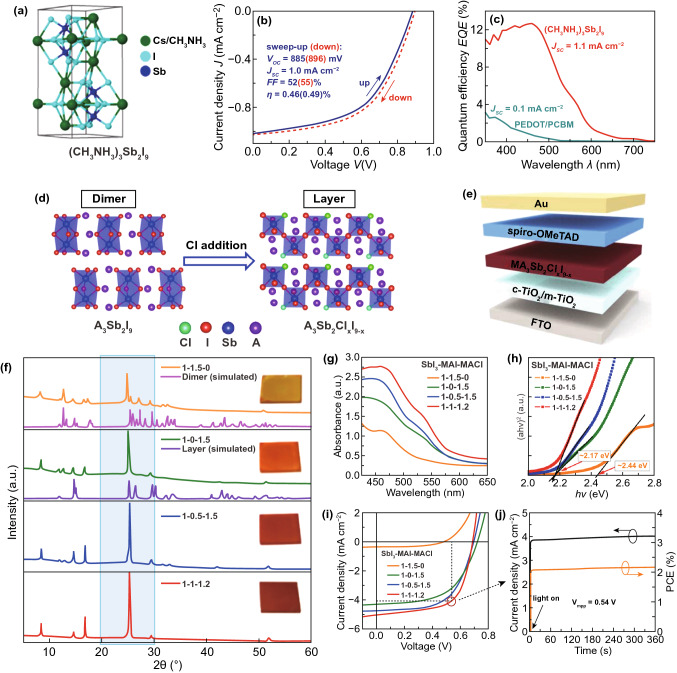
**a** Crystal structure of (CH_3_NH_3_)_3_Sb_2_I_9_ (space group P6_3_/*mmc*). **b** Illuminated *J*–*V* curves of (CH_3_NH_3_)_3_Sb_2_I_9_ solar cell measured with forward and backward scanning with a rate of 0.1 V s^−1^. **c** EQE measurement of the (CH_3_NH_3_)_3_Sb_2_I_9_ solar cell compared to the reference device of ITO/PEDOT/PCBM/ZnO-NP/Al. Reproduced with permission from Ref. [173] **d** Schematic plot of the Cl doping-induced transformation from the 0D dimer phase of A_3_Sb_2_I_9_ to the 2D layered phase of A_3_Sb_2_Cl_X_I_9−X_. **e** Schematic structure of the as-fabricated PSC. **f** XRD patterns of the films deposited from precursors containing SbI_3_, MAI, and MACl with molar ratios of 1:1.5:0, 1:0:1.5, 1:0.5:1.5, and 1:1:1.2. **g** Measured UV–vis absorbance spectra of the four types of films. **h** Tauc plots of the absorption coefficients for evaluating the bandgap values of the pure-iodine perovskites MA_3_Sb_2_I_9_ and Cl-containing mixed-halide perovskites MA_3_Sb_2_Cl_X_I_9−X_. **i** *J*–*V* curves for the devices fabricated with the four kinds of perovskite films. **j** Steady-state photocurrent output for the device based on 1-1-1.2 film at the maximum power point (red circle). Maximum power point voltage V_mpp_ is equal to 0.54 V. Reproduced with permission from Ref. [176]

Kirchartz et al. presented solution-treated trivalent antimony perovskite (CH_3_NH_3_)_3_Sb_2_I_9_ and fabricated a planar heterojunction solar cell with this compound (Fig. 14a–c), yielding a PCE of ~ 0.5%, a decent FF of 55%, a *V*_oc_ of 0.89 V and a low photocurrent density of 1.0 mA cm^−2^ [173]. They determined a peak absorption coefficient (α) of ~ 10^5^ cm^−1^ and an optical bandgap of 2.14 eV for amorphous (CH_3_NH_3_)_3_Sb_2_I_9_ films by photothermal deflection spectroscopy. The PL was observed at 1.58 eV, and the Urbach tail energy of this amorphous composite was 62 × 10^−3^ eV, demonstrating a substantial amount of energetic disorder [173]. The wide optical bandgap and energetic disorder may be the source of low photocurrent densities. In a word, this type of 0D dimer-phase trivalent antimony perovskite suffers from intrinsic problems including a low-symmetry induced indirect bandgap, strong quantum-confinement effect caused oversized gap values, and inferior hopping-like carrier transport, thus it is unfavorable for photovoltaic applications with extremely low PCEs of less than 0.5% obtained [62, 173].

The 2D layered phase can partially circumvent the above-mentioned problems and is expected to show a direct bandgap, smaller optical bandgap and good in-layer carrier transport properties. Recently, with the introduction of small-size Rb^+^ and NH_4_^+^ as the A-site cations, 2D layered phases of Rb_3_Sb_2_I_9_ [174] and (NH_4_)_3_Sb_2_I_9_ [175] have been realized using low-temperature solution processing. Zhou et al. performed comprehensive chemical composition engineering of the precursor solutions by incorporating MACl into the mixture of SbI_3_ and MAI, and successfully realized a phase transformation from the 0D dimer phase to the 2D layered one [176]. As displayed in Fig. 14d–j, the presence of the 2D layered phase was demonstrated by X-ray diffraction measurements, optical absorbance spectroscopy, and direct comparison of the experimental data with theoretical calculations. With high-quality films of the 2D layered MA_3_Sb_2_Cl_X_I_9−X_ perovskites as light absorbers, they fabricated solar cells with PCEs of over 2% [176].

So far, the Sb-based perovskites, similar to bismuth ones, still show a limited efficiency. But they own a bright application prospect once their nearly direct bandgap can be adjusted to a suitable value (e.g., doping) that matches with solar spectra. In addition, Sb-based perovskites can form a more stable 2D layered phase, which are better for carrier transport and display improved optoelectronic properties compared with the 0D Bi-based counterparts.

#### Chalcogen–Halogen Hybrid Perovskite

In 2015, Sun et al. proposed the anion-split approach based on first-principles calculation, where dual anions (e.g., halogen and chalcogen anions) were introduced while Pb was replaced by heterovalent non-toxic elements in order to keep the 3D perovskite structure and the charge neutrality (Fig. 8b, c) [52]. Figure 8b shows the atomic structure of MABiSeI_2_, which maintained the MAPbI_3_ tetragonal structure with a freely rotating MA^+^ ion according to ab initio molecular dynamics (AIMD) simulations.

Taking Bi as the core element of the B position, Shockley et al. examined a series of MABiChX_2_ (Ch = S, Se, Te; X = I, Br, Cl) compounds and found that CH_3_NH_3_BiSeI_2_ and CH_3_NH_3_BiSI_2_ exhibited improved optical absorption and reduced bandgaps (1.3–1.4 eV) over CH_3_NH_3_PbI_3_, which were proved to be the optimal value for solar cell absorbers according to the Shockley–Queisser theory (Fig. 8c) [177]. These results suggested that the 3D AB(Ch, X)_3_ perovskites represented by MABiSI_2_ could be promising candidates of photovoltaic absorbers once they were successfully synthesized. Unfortunately, the subsequent feasibility assessment of the proposed 3D AB(Ch, X)_3_ perovskites by Hong et al. via DFT calculations and solid-state reactions indicated that all of the as-proposed AB(Ch, X)_3_ perovskites are thermodynamically unstable [34], and they tend to decompose into ternary and/or binary phases or form nonperovskite phases. Recently, the work of Li et al. has proved the instability of chalcogen–halogen hybrid perovskite through experimental and computational analysis, and they found that AB(Ch, X)_3_ would be decomposed into a mixture of binary and ternary compounds (Sb_2_S_3_ and MA_3_Sb_2_I_9_) soon after being synthesized [178]. Upon inspection, the target perovskites are impossible to be successfully synthesized as designed. Finally, the researcher had to draw a conclusion that it may be a huge challenge to synthesize the proposed AB(Ch, X)_3_ perovskites due to their thermodynamic instability, which means there is still a long way for AB(Ch, X)_3_ perovskites to go for further applications in perovskite photovoltaic devices.

#### 0D Perovskite Derivative A_2_BX_6_

Previous analysis indicated that it is feasible to replace Pb of lead halide perovskites with tetravalent B(IV) substitutes. To accommodate the heterovalent substitute, the chemical formula needs to turn into A_2_BX_6_, which is derived from its ABX_3_ perovskite structure by removing half of B-site cations. Because of the large charge difference between them, the A_2_BX_6_ perovskite variant is sometimes referred to as the A_2_B□X_6_-type vacant ordered double perovskite [86, 179–181]. The B-site vacancies (denoted as □) and the remaining B-site cations generally adopt a rock salt arrangement in the A_2_BX_6_ perovskite structure. Due to the absence of connectivity between the [BX_6_] octahedra, the A_2_BX_6_ perovskite variants are actually 0D nonperovskites despite researchers still would like to call them perovskites. The optoelectronic properties of the A_2_BX_6_-type perovskite materials are rather different from those of 3D ABX_3_ (B = Pb, Sn, and Ge) perovskites due to the isolated [BX_6_] octahedra in A_2_BX_6_ compounds. Among the A_2_BX_6_ compounds [182–185], A_2_SnI_6_ (A = Cs, MA) [182, 183] and Cs_2_TiBr_6_ [181, 182] have been investigated for photovoltaic applications. These A_2_BX_6_ compounds show intrinsic n-type conductivity. Compared to 3D CsSnI_3_ with better defect tolerance, the A_2_BX_6_-type materials with I vacancies and Sn interstitial defects have deeper levels in their bandgaps owing to the strong covalent nature of the [SnI_6_] octahedron, seriously impacting optoelectronic performances of devices.

The vacancy-ordered A_2_M(IV)X_6_ double perovskites was found to have suitable direct bandgaps. In 2016, Cao et al. found the spontaneous oxidative conversion of unstable CsSnI_3_ to air-stable Cs_2_SnI_6_ in air [183]. The Cs_2_SnI_6_ perovskite was adopted as the light absorber layer of lead-free perovskite solar cell for the first time due to its small bandgap of 1.48 eV and high absorption coefficient, showing a PCE of about 1% with a *V*_oc_ of 0.51 V and a *J*_sc_ of 5.41 mA cm^−2^ after optimizing the perovskite film thickness.

Soon after, Cao et al. synthesized Cs_2_SnI_6_ powder with a modified solution process and fabricated mesoporous solar cells [183]. They achieved a PCE of 0.96% by carefully controlling ZnO nanorod length and pore size. Chang et al. reported a new material Cs_2_SnI_6−x_Br_x_ with mixed halides which provided desired bandgaps of ~ 1.3 to ~ 2.9 eV suitable for solar cells via simple tune of Br component. They applied this perovskite material as light absorbing layer to PSCs and achieved an optimal conversion efficiency of 2.1% as x = 2 [184]. Recently, Cs_2_TiBr_6_ thin films were prepared through a facile low-temperature vapor-based method and then incorporated into planar heterojunction PSCs [185]. Subsequently, Chen et al. also demonstrated that the Cs_2_TiBr_6_ thin film has a suitable bandgap of 1.8 eV, a long and balanced carrier diffusion length, and an appropriate energy level, with which solar cells produced an efficiency of up to 3.3% [186].

#### Double Perovskite A_2_B(I)B(III)X_6_

**Fig. 15:**
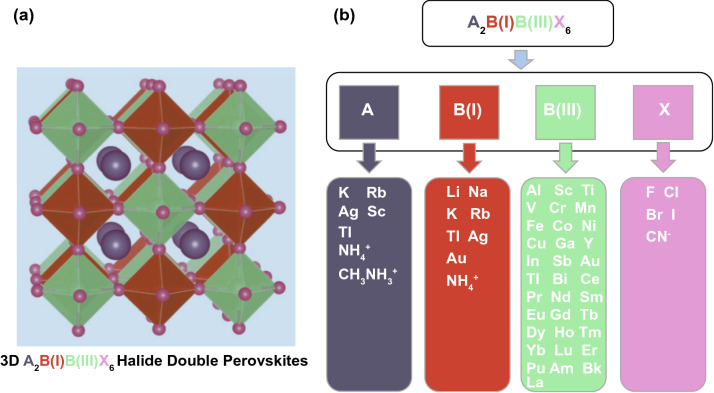
Crystal structure of double perovskite A_2_B(I)B(III)X_6_ (**a**) and the elements/functional groups that can form double perovskite A_2_B(I)B(III)X_6_ (**b**)

Lead-free halide double perovskites materials were known as “elpasolites” many years ago, whose name was from the mineral K_2_NaAlF_6_. In 2016, Giustino et al. counted the elements belonging to halide elpasolites [27]. As shown in Fig. 15, it can be clearly seen that 7 elements can occupy the A-site, and 8 elements including NH_3_^+^ can occupy the B(I) site and 34 elements can occupy the B(III) site, while 5 elements containing the cyanide CN^−^ are able to occupy the X site. Although many of these compounds are available, it requires to satisfy some prerequisites for photovoltaic applications, such as suitable bandgap, high carrier mobility, low defect state and good thermodynamic stability. Through the element selection of B(I) site and B(III) site in the double perovskite, the bandgap can be well adjusted and thus exhibits excellent optoelectronic properties. For example, Cs_2_NaBiI_6_ has a direct bandgap of 1.66 eV. Through element adjustment, Cs_2_AgInBr_6_ with a direct bandgap of 1.50 eV and Cs_2_InSbCl_6_ and Cs_2_InBiCl_6_ with a direct bandgap of about 1.0 eV can be obtained. At present, the researchers have discovered the double perovskite material Cs_2_AgBiBr_6_ possesses with a suitable bandgap of 1.95 eV, which can be used as light absorber in solar cells. To date, Cs_2_AgBiBr_6_ is the most commonly used photovoltaic material in lead-free halide double perovskite-based solar cells [187–190]. Bein et al. fabricated Cs_2_AgBiBr_6_ films with a spin coating method and incorporated them into solar cells for the first time in 2017 [187]. After optimizing synthesis conditions, the Cs_2_AgBiBr_6_-based solar cells without encapsulation showed a PCE of 2.43%, a *V*_oc_ of exceeding 1 V and excellent stability in air. The fly in the ointment is that the device has a serious hysteresis effect.

In 2018, Xiao et al. prepared a transparent solution of Cs_2_AgBiBr_6_ in DMSO and fabricated the Cs_2_AgBiBr_6_ film using spin coating technique by low-pressure-assisted solution processing under ambient conditions [188]. Differently from the conventional annealing method, the spin-coated film was quickly moved to a low-pressure chamber pumped to 20 Pa, where the transparent film gradually turned to light yellow. A smooth morphology was obtained compared with the rough films prepared by conventional annealing methods. Finally, planar heterojunction solar cells were manufactured with optimized Cs_2_AgBiBr_6_ films and P3HT hole transport layer, and a PCE of 1.44% was achieved.

**Fig. 16:**
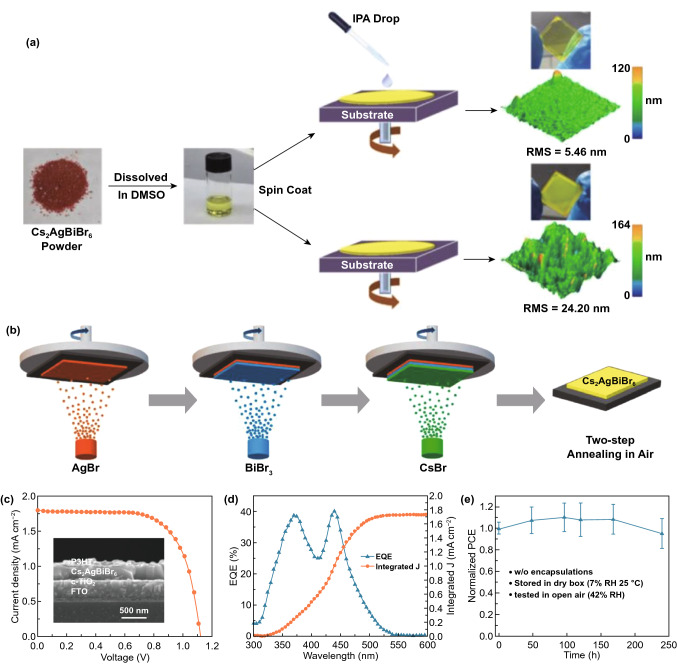
**a** Schematic illustration of the spin coating process of Cs_2_AgBiBr_4_ with and without antisolvent dropping program, and the morphology of the as-prepared film. Reproduced with permission from Ref. [189] **b** Scheme of sequential vapor deposition process. **c** *J*–*V* curve of the optimized solar cell. Inset: Cross-sectional SEM image of the device. **d** EQE spectrum and the integrated current density with AM 1.5G photon flux. **e** Evaluation of long-term stability of the solar cells stored in dry box. Reproduced with permission from Ref. [191]

In a subsequent work, Wu et al. used antisolvent dropping method and post-annealing process to realize high-quality Cs_2_AgBiBr_6_ films with ultra-smooth morphology, microsized grains, and high crystallinity (Fig. 16a). Then they fabricated inverted planar heterojunction solar cells with the as-prepared Cs_2_AgBiBr_6_ films and got a high PCE of 2.23% [189]. It should be noted that the *J*–*V* hysteresis was clearly showed in the above-mentioned two works, which was closely related with the large amount of defects in Cs_2_AgBiBr_6_ films. Grancini et al. precisely controlled the perovskite deposition parameters to obtain a highly uniform and compact Cs_2_AgBiBr_6_ film, and modified the device interfaces with small molecular/polymeric hole-transporting materials to eliminate the hysteresis effect [190].

Recently, Liu et al. successfully prepared double perovskite Cs_2_AgBiBr_6_ thin films through a sequential vapor deposition procedure (Fig. 16b) [191]. The as-fabricated thin films with pure double perovskite phase showed large grain sizes, uniform and smooth surface morphology along with a PL lifetime of 117 ns, indicative of a significant potential in photovoltaic applications. The solar cells with planar device structure obtained an optimal PCE of 1.37%, which maintained 90% of the initial efficiency after 240 h storage under ambient condition (Fig. 16c–e). In addition to Cs_2_AgBiBr_6_, Cs_2_NaBiI_6_ has also been used as a light absorbing layer for PSCs and the device showed a good stability despite of a low efficiency of only 0.42% [192]. Relevant results for most types of double PSCs based on various device architectures are summarized in Table 3 [187–192].

**Table 3:** Photovoltaic parameters of PSCs based on double perovskite A_2_B(I)B(III)X_6_ absorbers

Absorber	Method for preparing perovskite film	Device architecture	*V*_oc_ (V)	*J*_sc_ (mA cm^−2^)	FF (%)	PCE (%)	References
Cs_2_AgBiBr_6_	Spin coating	c-TiO_2_/m-TiO_2_/perovskite/spiro-MeOTAD/Au	0.98	3.93	63	2.43	[187]
Cs_2_AgBiBr_6_	Low-pressure-assisted solution processing under ambient conditions	SnO_2_/Cs_2_AgBiBr_6_/P3HT/Au	1.04	1.78	78	1.44	[188]
Cs_2_AgBiBr_6_	Antisolvent dropping technology and post-annealing process	Cu-NiO/Cs_2_AgBiBr_6_/C_60_/BCP/Ag	1.00	3.23	68.4	2.21	[189]
Cs_2_AgBiBr_6_	Sequential vapor deposition method	c-TiO_2_/perovskite/P3HT/Au	–	–	–	1.37	[191]
Cs_2_AgBiBr_6_	Spin coating deposition and annealing at high temperature	c-TiO_2_/m-TiO_2_/perovskite/PTAA/Au	1.02	1.84	67	1.26	[190]
Cs_2_AgBiBr_6_	Spin coating deposition and annealing at high temperature	c-TiO_2_/m-TiO_2_/perovskite/PCPDTBT/Au	0.71	1.67	57	0.68	[190]
Cs_2_AgBiBr_6_	Spin coating deposition and annealing at high temperature	c-TiO_2_/m-TiO_2_/perovskite/spiro-MeOTAD/Au	0.64	2.45	57	0.90	[190]
Cs_2_NaBiI_6_	Spin coating	c-TiO_2_/m-TiO_2_/perovskite/spiro-MeOTAD/Au	0.47	1.99	44	0.42	[194]

At the initial design stage of lead-free halide double perovskite materials, researchers specifically emphasized the need to ensure the thermodynamic stability of the material [186–188]. Therefore, the halide double perovskite materials used in solar cells show excellent stability, which will be an outstanding advantage in practical application. In addition, the optical bandgap and other optoelectronic properties of the double perovskites can be optimized through the use of both B(I) and B(III) elements [186–194]. Thus, these characteristics make the halide double perovskite a good candidate for high-performance, stable and environmentally friendly solar cells.

## Conclusion and Prospect

In this review, we focused our attention on environmentally friendly lead-free perovskite materials and combed the related progress on theoretical and experimental works. Herein, all of lead-free perovskites consisting of Sn (II), Cu (II), Bi(III), Sb (III), Sn (IV), Ti (IV), and Ag(I)Bi(III) were introduced from the isovalent and heterovalent elements replacement perspectives. We summarized the material preparation, device performance and stability of each lead-free perovskite materials and presented their possible issues and potential development prospects. For Sn-based perovskites, this type of material has a relatively high absorption coefficient (close to 1.80 × 10^4^ cm^−1^), but the easy oxidation of Sn^2+^ to Sn^4+^ is also considered to be the main problem limiting the development of Sn-based PSCs. Similar to Sn-based perovskites, Ge-based perovskites also face the trouble of being easily decomposed by oxidation. In addition, compared with Sn^2+^, Bi and Sb are very stable when they are in the +3 valence state in the atmosphere. However, the efficiency of Bi- and Sb-based PSCs is still very low. Further optimization of their bandgaps as well as film morphology, manufacture methods and device structures are feasible solutions to further improve device performance. The transition metal perovskites represented by Cu-based perovskites also need to reduce their wide optical bandgap and improve their charge transporting properties to develop more efficient PSCs. Besides, the application of AB(Ch, X)_3_ perovskites for PSCs is facing great challenges due to the thermodynamic instability. In addition to the above-mentioned materials, the halide double perovskite has attracted people’s attention because of its excellent stability and bandgap tunability.

In summary, we have reviewed the past experimental work and comprehensively understood the development and application prospects of current lead-free perovskite materials. Lead-free perovskites enable to avoid a high content of the toxic, polluting and bioaccumulative lead hazards and are more conducive to commercial production and application. At the same time, theoretical simulations have made important contributions to comprehending the fundamental physics behind the efficiency, stability, and carrier transport properties of lead-free PSCs. Using scientific theoretical calculations and reasonable solar cell preparation methods, we believe that the research on lead-free PSCs will soon have a clear breakthrough. Here, we are looking forward to the further development of lead-free perovskite materials and PSCs. Finally, we expect that the performance as well as the stability of lead-free perovskites, especially 2D layered perovskites, can be continuously improved to meet the needs of commercial development. In spite of slower development of lead-free perovskites than lead ones, we believe that the efficiency based on lead-free perovskite materials can break through 15% after further in-depth study, and we should keep warm toward this research.
